# Tannic Acid-Modified Silver and Gold Nanoparticles as Novel Stimulators of Dendritic Cells Activation

**DOI:** 10.3389/fimmu.2018.01115

**Published:** 2018-05-22

**Authors:** Piotr Orlowski, Emilia Tomaszewska, Katarzyna Ranoszek-Soliwoda, Marianna Gniadek, Olga Labedz, Tadeusz Malewski, Julita Nowakowska, Grzegorz Chodaczek, Grzegorz Celichowski, Jaroslaw Grobelny, Malgorzata Krzyzowska

**Affiliations:** ^1^Military Institute of Hygiene and Epidemiology, Warsaw, Poland; ^2^Department of Materials Technology and Chemistry, Faculty of Chemistry, University of Lodz, Lodz, Poland; ^3^Faculty of Chemistry, University of Warsaw, Warsaw, Poland; ^4^Museum and Institute of Zoology, Polish Academy of Science, Warsaw, Poland; ^5^Laboratory of Electron and Confocal Microscopy, Faculty of Biology, University of Warsaw, Warsaw, Poland; ^6^Wroclaw Research Centrum EIT+, Wroclaw, Poland

**Keywords:** silver nanoparticles, gold nanoparticles, dendritic cells, HSV-2, tannic acid

## Abstract

Silver nanoparticles (AgNPs) are promising new antimicrobial agents against a wide range of skin and mucosal pathogens. However, their interaction with the immune system is currently not fully understood. Dendritic cells (DCs) are crucial during development of T cell-specific responses against bacterial and viral pathogens. We have previously shown that tannic acid-modified silver nanoparticles (TA-AgNPs) consist of a promising microbicide against HSV-2. The aim of this study was to compare the ability of TA-AgNPs or TA-AuNPs of similar sizes (TA-Ag/AuNPs) to induce DCs maturation and activation in the presence of HSV-2 antigens when used at non-toxic doses. First, we used JAWS II DC line to test toxicity, ultrastructure as well as activation markers (MHC I and II, CD40, CD80, CD86, PD-L1) and cytokine production in the presence of TA-Ag/AuNPs. Preparations of HSV-2 treated with nanoparticles (TA-Ag/AuNPs-HSV-2) were further used to investigate HSV-2 antigen uptake, activation markers, TLR9 expression, and cytokine production. Additionally, we accessed proliferation and activation of HSV-2-specific T cells by DCs treated with TA-AgNP/AuNPs-HSV-2. We found that both TA-AgNPs and TA-AuNPs were efficiently internalized by DCs and induced activated ultrastructure. Although TA-AgNPs were more toxic than TA-AuNPs in corresponding sizes, they were also more potent stimulators of DCs maturation and TLR9 expression. TA-Ag/AuNPs-HSV-2 helped to overcome inhibition of DCs maturation by live or inactivated virus through up-regulation of MHC II and CD86 and down-regulation of CD80 expression. Down-regulation of CD40 expression in HSV-2-infected DCs was reversed when HSV-2 was treated with TA-NPs sized >30 nm. On the other hand, small-sized TA-AgNPs helped to better internalize HSV-2 antigens. HSV-2 treated with both types of NPs stimulated activation of JAWS II and memory CD8+ T cells, while TA-AgNPs treatment induced IFN-γ producing CD4+ and CD8+ T cells. Our study shows that TA-AgNPs or TA-AuNPs are good activators of DCs, albeit their final effect upon maturation and activation may be metal and size dependent. We conclude that TA-Ag/AuNPs consist of a novel class of nano-adjuvants, which can help to overcome virus-induced suppression of DCs activation.

## Introduction

Nanoparticles (NPs) are increasingly recognized for their potential applications in cosmetics, pharmaceuticals, and medicine (1–5). The small size of NPs enables them to infiltrate tissues including lymphoid tissues and subsequently target immune cells, which makes them potentially useful in immunotherapies (6). Conjugation of NPs with biologically active compounds may further lead to new advantageous properties, better bio-distribution, and bioactivity.

Dendritic cells (DCs) induce immunity (cellular and humoral) through response to foreign pathogens by activation of cellular immune response. In the periphery, DCs exist in immature state and are activated and differentiated into mature DCs through the recognition of antigens (7, 8). After antigen capture, DCs acquire a mature phenotype, lose their endocytic and phagocytic receptors, and migrate to lymphoid organs for T cells priming (7, 8). Taking into account the fact that DCs are present throughout the body, they consist, together with other mechanisms of the adaptive immune response, the first line of encounter of the immune system with NPs.

Metallic NPs (MeNPs) are relatively non-biodegradable, have rigid structures, and possess simple synthesis methodology. Different MeNPs (gold, silver, and nickel) have been used in conjunction with several Ag for distinct microorganisms and showed the ability to generate humoral and cytotoxic responses (9–12). A number of reports have indicated that gold NPs (AuNPs) facilitate delivery of antigens and adjuvants to the immune system, promote the therapeutic effect, and possess an adjuvant effect on their own (9–11). AuNPs accumulate in DCs and B cells of the spleen after intravenous injection (6). Furthermore, AgNPs also show a significant adjuvant effect mainly due to the recruitment and activation of local leukocytes, especially of macrophages (13). On the other hand, DCs exposed to silver NPs (AgNPs) show a decrease in cell viability and production of reactive oxygen species (ROS) but no production of inflammatory cytokines such as TNF-α or IL-1β (14).

In our previous work, we demonstrated antiviral properties of tannic acid modified-AgNPs (TA-AgNPs) both *in vitro* and *in vivo* using the murine model of intravaginal HSV-2 infection (15). The antiviral mechanism of TA-AgNPs involved blocking of virus attachment, entry, and induction of anti-viral cytokine and chemokine production. Cytokine and chemokine production during HSV-2 infection showed time and size-related differences for treatment with each NP type (15). Furthermore, TA-AgNPs also showed size-dependent immunomodulatory properties against uninfected monocytes and keratinocytes (16, 17).

Herpes simplex virus (HSV) causes a contagious infection that affects approximately 60% to 95% of adults worldwide. HSV-1 is associated mainly with infections of the mouth, pharynx, face, eye, and central nervous system (CNS), while HSV-2 is associated with infections of the anogenital region. HSV-1 and -2 persist in the body by becoming latent in the cell bodies of nerves and after the initial or primary infection (18). Currently, the only way of herpes treatment is the use of antiviral drugs, blocking viral replication. Given the sub-optimal performance of HSV vaccine candidates to date and the role of DCs in priming cellular responses, a more directed approach specifically targeted at DCs may be required to improve vaccine efficacy (19). One possible solution is to target DCs with appropriate antigens/adjuvants. Since NPs possessing anti-viral and immunomodulatory activities can be engulfed by DCs and used as “antigen delivery/enhancement system,” they can also play a role of the locally applied adjuvants (20, 21).

In the present study, we showed how differently sized TA-AgNPs and -AuNPs, applied at the non-toxic concentrations, influence maturation of JAWS II mouse DCs line, production of cytokines, and expression of TLR9. Furthermore, we showed that NPs treatment of HSV-2 can overcome inhibited maturation of DCs, increase antigen uptake as well as activation of HSV-2 specific memory T cells as well as INF-γ producing CD4+ and CD8+ T cells.

## Materials and Methods

### Ethics Statement

This study was performed in strict accordance with the recommendations of the Polish Act of 21 January 2005 on animal experiments (OJ no. 33, item 289) and Directive 2010/63/EU of the European Parliament and the Council of 22 September 2010 on the protection of animals used for scientific purposes. The protocol was approved by the 4th Local Committee on the Ethics of Animal Experiments in Warsaw, Poland (Permit Number: 51/2013).

### Synthesis of AuNPs and AgNPs

#### Materials and Methods of Synthesis

Gold (III) chloride hydrate (HAuCl_4_·H_2_O, Sigma-Aldrich, St. Louis, MO, USA, ≥ 49% Au basis), silver nitrite (AgNO_3_; Sigma-Aldrich, 99.999% metal basis), sodium citrate (C_6_H_5_Na_3_O_7_·2H_2_O, Sigma-Aldrich, ≥99%), ammonium citrate tribasic (C_6_H_17_N_3_O_7_, Sigma-Aldrich, ≥ 97%), tannic acid (C_76_H_52_O_46_, Fluka, Seelze, Germany), and sodium borohydride (NaBH_4_, Fluka, ≥99%) were used without additional purification. For all experiments, deionized water was obtained from Deionizer Millipore Simplicity UV system (specific resistivity of water was 18.2 MΩ cm, Millipore, Merck, Warsaw, Poland). All AuNPs and AgNPs colloids were stored at darkness and filtered through a 0.1 μm polyvinylidene fluoride (PVDF) membrane before use in biological tests.

Synthesized NPs were characterized using scanning transmission electron microscopy (STEM), dynamic light scattering (DLS), and UV-Vis spectroscopy. STEM measurements were performed using scanning electron microscope Nova NanoSEM 450, accelerating voltage of 30 kV (FEI, Hillsboro, OR, USA) equipped with a detector for transmitted electron acquisition (STEM II). Samples for STEM were prepared as follows: a drop of colloid was deposited onto a carbon-coated copper grid (300 mesh) and left for solvent evaporation under ambient conditions. The DLS and Zeta potential measurements were carried out using Nano ZS Zetasizer system (Malvern Instruments, Malvern, Great Britain) with the He−Ne laser (633 nm) as the light source (scattering angle 173°, measurement temperature 25°C; medium viscosity 0.887 mPa⋅s, material refractive index 1.330). DLS measurements were performed in disposable quartz cuvettes and Zeta potential measurements in disposable folded capillary zeta cells (DTS 1070). For analysis of Zeta potential measurements, the Smoluchowski model was applied (22). The UV−vis spectra were recorded with the spectrophotometer USB2000 + detector (miniature fiber optic spectrometer) Ocean Optics, HL-2000 (tungsten halogen light sources) using quartz cuvettes (Ocean Optics, Winter Park, FL, USA).

#### Gold Nanoparticles

AuNPs with the size of 10 nm, 34 nm, and 62 nm and weight concentration of Au in colloid equal to 100 ppm were synthesized in water by reduction of gold (III) chloride hydrate. The synthesis procedures were described in our previous work (17). Briefly, an aqueous solution of gold (III) chloride hydrate was boiled and vigorously stirred under reflux. Next, a mixture of aqueous solutions of sodium/ammonium citrate and/or tannic acid was added into the solution. The amounts of reagents used for syntheses are summarized in Table 1. After the reducing mixture changed color to red indicating the formation of AuNPs, the colloids were stirred for additional 15 min under reflux and cooled down to a room temperature.

**Table 1:** The amounts of reagents used for the syntheses of AuNPs.

Size of AuNPs	Gold (III) chloride hydrate	Reducing mixture
10 nm	94.4 g, 0.018 wt%	Sodium citrate (3.9 g, 1 wt%) Tannic acid (1.7 g, 1 wt%)
34 nm	96.2 g, 0.018 wt%	Tannic acid (3.8 g, 5 wt%)
62 nm	97.8 g, 0.018 wt%	Tribase ammonium citrate (2.2 g, 2 wt%)

#### Silver Nanoparticles

Silver nanoparticles (100 ppm) with the size of 10 nm, 37 nm, and 59 nm were synthesized in water by the chemical reduction method. The synthesis procedures were described previously (17) and were as follows: a reducing mixture was added to the aqueous solution of silver nitrite heated to the boiling point under reflux (except for AgNPs 10 nm, where the aqueous solution of silver nitrite at room temperature was used). The amounts of reagents used for syntheses are summarized in Table 2. After a few minutes, the color of the solution changed to brownish indicating the formation of AgNPs. The mixture was stirred for additional 15 min.

**Table 2:** The amounts of reagents used for the syntheses of AgNPs.

Size of AgNPs	Silver nitrite	Reducing mixture
10 nm	94.5 g, 0.0166 wt%	Tannic acid (0.6 g, 5 wt%) Sodium citrate (4.2 g, 4 wt%) Sodium borohydride (0.7 g, 2 wt%)
37 nm	95.2 g, 0.0165 wt%	Tannic acid (0.6 g, 5 wt%) Sodium citrate (4.2 g, 4 wt%)
59 nm	97.60 g, 0.0161 wt%	Tannic acid (1.3 g, 5 wt%) Sodium citrate (1.1 g, 4 wt%)

### Virus

HSV-2 strain 333 was grown and titrated (PFU/ml) in African green monkey kidney cells (GMK-AH1). The heat-inactivated virus was prepared by heating the virus suspended in a complete cell culture medium at 56°C for 60 min. For UV inactivation, virus suspension was exposed in sterile conditions to UV lamp for 45 min on ice. No viral particles were detected by plaque assay in the supernatants of cell cultures treated with UV- or heat-inactivated HSV-2. For inactivation of HSV-2 with NPs, a virus inoculum was pre-incubated with 2.5 µg/ml of silver or AuNPs for 1 h, as described previously (15).

### Cell Lines, Bone-Marrow Derived DCs (BMDCs), and Treatment With NPs

African green monkey kidney cells (GMK-AH1) were a gift from the Swedish Institute for Infectious Disease Control, Stockholm, and were cultured in MEM alpha modification (α-MEM) supplemented with 10% heat inactivated fetal bovine serum (HI-FBS), 100 U/ml penicillin, and 100 µg/ml streptomycin B (Gibco by Thermo Fisher Scientific, MA, USA) in standard conditions (37°C, 5% CO_2_). The immature DCs (JAWSII) were purchased from American Type Culture Collection (ATCC, CRL-11904, Rockville, MD, USA) and were grown in α-MEM with deoxy- and ribonucleosides, supplemented with 20% HI-FBS, 4 mM l-glutamine, 1 mM sodium pyruvate, 100 U/ml penicillin, 100 µg/ml streptomycin (Gibco), and 5 ng/ml murine granulocyte macrophage colony-stimulating factor (GM-CSF) (Sigma-Aldrich), in standard conditions.

JAWSII cells were seeded into 24-well plates at a density of 5 × 10^4^/ml for 24 h before exposure to NPs at the concentration from 0.5 to 10 µg/ml or infection with HSV-2, heat inactivated HSV-2, or HSV-2 inactivated with 2.5 µg/ml NPs at the multiplicity of infection (MOI) 0.5–1. After another 24 h, the cells were used for further analyses. Lipopolysaccharide (LPS, Sigma-Aldrich), a TLR-4 ligand, or polyinosinic-polycytidylic acid potassium salt (Poly I:C) (Sigma-Aldrich), a TLR-3 ligand, at the concentration of 10 µg/ml and 5 µg/ml, respectively, were used as positive control.

To inhibit NPs uptake in some experiments, the following inhibitors were used for 1 h: 10 µg/ml monodansyl cadeverine (MDC), 200 µM genistein, 10 µg/ml colchicine, 400 nM wortmannin, and 5 µg/ml cytochalasin D (CChD, Sigma-Aldrich), then washed out. Subsequently, the cells were exposed to 2.5 µg/ml NPs for 6 h. Concentrations of inhibitors were chosen on the basis of lack of toxic effects measured by tests described below.

Primary cultures of BMDCs were prepared by culturing bone marrow cells isolated from C57BL6 mice in the D-MEM (Gibco) culture medium supplemented with 25 ng/ml GM-CSF and 15 ng/ml IL-4 (Gibco), 4,500 mg/l glucose, antibiotics (penicillin and streptomycin), l-glutamine, 10% fetal bovine serum (Gibco) for 5 days. At day 5, primary cultures of DCs were washed and subjected to treatment, as described above. All experiments were performed at least in triplicates.

### Toxicity Assessment

To determine cell apoptosis or necrosis, Apoptosis Detection kit was used (Beckton Dickinson, Franklin Lakes, NJ, USA). The kit distinguishes apoptotic from necrotic cells by detecting apoptotic asymmetry of the cell membrane with annexin V-FITC, while necrotic cells are labeled with red-fluorescent propidium iodide (PI). Briefly, after exposure to NPs, cells were collected, washed with PBS, and then suspended in staining solution containing 5 µg/ml of annexin V and 1 µg/ml of PI, incubated for 10 min in 4°C, and analyzed in FACS Calibur using CellQuest programme (Beckton Dickinson). Apoptotic cells were defined as the percentage of annexin V-FITC positive cells, while necrotic cells were defined as the percentage of PI positive cells.

To assess the mitochondrial potential after NPs treatment, the cells were stained with 5,5′,6,6′-tetrachloro-1,1′,3′-teraethyl-benzimidazolylcarbocyanine iodide (JC-1, Sigma-Aldrich). For staining, cells were harvested and incubated with α-MEM supplemented with 20% FBS and 5 µg/ml JC-1 at 37°C for 15 min. Cells were then washed two times in PBS and analyzed in FACS Calibur using CellQuest program (Beckton-Dickinson) for the percentage of cells with a decrease in red to green fluorescence intensity ratio. The Neutral red test was performed as described previously (16). The viability of cells was expressed as a percentage of the control, untreated cells (100%).

### Transmission Electron Microscopy (TEM) and Scanning Electron Microscopy (SEM) Imaging

For TEM imaging of JAWS II ultrastructure, cells were collected, fixed, and processed as described previously (17). Ultrathin sections (70–90 nm) were cut on a Ultracut E microtome (Reichert-Jung, Austria). The images were acquired using Zeiss Libra 120 transmission electron microscopy (Zeiss, Jena, Germany).

Scanning electron microscopy images of the cells surfaces were obtained by incubating JAWSII with 5 µg/ml of NPs for 3 h, then the cells were fixed with warm 2.5% glutaraldehyde in 0.1 M phosphate buffer pH 7.2 for 20 min, as described in Ref. (17) and acquired using FE-SEM Merlin (Zeiss, Jena, Germany).

### Confocal Imaging

For microscopic detection of NPs, cells were plated on slides at a density of 1 × 10^5^/ml for 18 h before exposure to NPs. After 24 h of exposure to 5 µg/ml of TA-AgNPs or TA-AuNPs, the medium was discarded and the cells were fixed with 4% paraformaldehyde (PFA) in PBS (Sigma-Aldrich), washed twice with PBS and stained with LysoTracker™ Red DND-99 (Thermo Fisher Scientific) according to the manufacturer′s protocol. The images were captured on an upright Leica SP8 resonant scanning confocal system (Leica Microsystem, Wetzlar, Germany). Stacks of confocal 8-bit images with a pixel size of 0.186 µm and a 0.5 µm Z step were acquired using 40× oil immersion objective (NA 1.30). The pinhole was set to 1 AU. Nanoparticles were visualized in a reflection mode using a 638 nm laser line. DNA signal from Hoechst 33342 was excited using a 405 nm laser line and 460–490 nm emission range was collected. Lysosomes were stained in live cells with LysoTracker Red DND-99 (excitation with a 552 nm laser line and recorded emission was 561–618 nm). The acquisition was performed in a sequential mode. Images showing equatorial optical sections were analyzed in Fiji/ImageJ software (National Institutes of Health, USA), and Manders’ Colocalization Coefficients were calculated in thresholded images.

### Flow Cytometry Phenotypic Analysis, High Content Screening (HCS), and Measurement of Cytokines

After treatments, cells were collected, washed with PBS containing 2% FBS, and blocked with anti-mouse CD16/CD32 (clone 93; eBioscience, San Diego, CA, USA) on ice for 10 min. After washing, cells were stained using anti-MHC I-FITC (clone 34-1-2 S; eBioscience), anti-CD80-PE (clone 16-10 A1; eBioscience), anti-CD40-FITC (clone HM40-3; eBioscience), anti-MHC II-PE (clone M5/114.15.2; eBioscience), anti-CD86-FITC (clone GL1; eBioscience), anti-PD-L1-PE (clone MIH5; eBioscience), anti-CD3e-Alexa Fluor 488 (clone 145-2C11; eBioscience), anti-CD4-PE (clone GK1.5; eBioscience), anti-CD8-BV421 (clone 53-6.7; Becton Dickinson), anti-CD44-APC (clone IM7; eBioscience), and anti-CD69-APC (clone H1.2F3; eBioscience). Following the immunolabelling for the extracellular markers, cells were fixed with Perm/Wash buffer (BD Bioscience) and were incubated with anti-IFN-γ APC-Cy7 (clone-XMG1.2; eBioscience). The stained cell suspensions were analyzed in FACS Calibur or FACS Verse for the percentage of positively stained cells or the mean fluorescence intensity.

For measurement of HSV-2 antigens incorporated 6 h post infection (p. i.), we used HCS. After infection, cells were fixed with acetone–methanol mixture, air dried, and kept until antigen detection at −20°C. After rehydration in PBS, cells were stained with FITC-conjugated anti-HSV-1/HSV-2 polyclonal antibody (Dako, Glostrup, Denmark). Nuclei were stained with Hoechst 33342 (Sigma-Aldrich). Images were obtained and analyzed by ArrayScan™ XTI High Content Platform equipped with HCS Studio™ 2.0 Cell Analysis Software (Thermo Fisher Scientific).

We used Cytometric Bead Array (CBA) Mouse Th1/Th2/Th17 Kit (IL-10, IL-17A, TNF, IFN-γ, IL-6, IL-4, IL-2) (Beckton Dickinson) to measure cytokines from culture supernatants by flow cytometry according to the manufacturer′s protocol. The results are presented as means of assays performed in triplicates. Data were analyzed using FCAP 0.1 BD Cytometric Bead Array and BD Array 1.4 software assay (Beckton Dickinson).

### TLR9 mRNA Levels

Total RNA was extracted with the Universal RNA Purification Kit (Eurx, Gdansk, Poland), following the manufacturer′s protocol. The amount of total RNA extracted and its purification from protein and polysaccharides was determined with a NanoDrop 2000 spectrophotometer (Thermo Fisher Scientific). RNA integrity was verified electrophoretically in a 1.5% agarose gel stained with ethidium bromide. Only samples that satisfied both the quality and integrity requirements were used in subsequent experiments. Three high-quality RNA samples (i.e., biological replicates) were obtained for each condition. Reverse transcription was carried out using Enhanced Avian HS RT-PCR Kit (Sigma-Aldrich), in line with the manufacturer′s instructions. To analyze expression of tlr9 gene, we used the primers 5′GCCACATTCTATACAGGGATTGG3′ and 5′GCCACATTCTATACAGGGATTGG3′, Gapdh was used as a reporter gene in our experiment. Real-time PCRs were performed in the RotorGene 6000 system (Qiagen, Hilden, Germany). Reactions were carried out using LuminoCt SYBR Green qPCR Master Mix (Sigma-Aldrich), while cycle threshold (Ct) estimates were obtained using the relative quantification module in the software package. Fluorescence data were analyzed subsequently using the Tm calling module in the RotorGene 6000 software. The 2^ΔΔCt^ method was used in calculating the relative ratio, but instead of value 2, the correct amplification efficiency was used (23). We used a noise-resistant iterative nonlinear regression algorithm (Real-time PCR miner; www.miner.ewindup.info) to determine the efficiency of the PCR reaction (24). Levels of mRNA were counted from three PCR reactions for each sample.

### Mice Challenge and T-Cells Activation Assay

Female C57BL/6 mice were challenged every 30 days subcutaneously with 2.5 × 10^4^ PFU of UV inactivated HSV-2. After 240 days, and two weeks after the last challenge, the mice were sacrificed and spleens were isolated to obtain single cell suspensions. T-lymphocytes were isolated using MACS Pan-T Cell Isolation Kit according to the manufacturer′s protocol (Miltenyi Biotec, Surrey, UK). The isolated T cells were stained with carboxyfluorescein succinimidyl ester (CFSE) (Sigma-Aldrich). Briefly, cells washed with PBS were incubated with 5 µM CFSE, then washed twice with 2% FBS/PBS. JAWSII were seeded for 24 h in U-bottomed 96-plates at the density of 1 × 10^4^/well and infected with HSV-2, heat-inactivated HSV-2 or exposed to HSV-2 treated with NPs as described above. Twenty-four hour post-infection/treatment medium was replaced to RPMI supplemented with 10% HI-FBS, 100 U/ml penicillin, 100 µg/ml streptomycin (Gibco), and then co-cultured with 10^5^/well T cells. After 72 h, T cells proliferation was analyzed in FACS Calibur as described above.

### T Cell Proliferation Assay

T cell proliferation was measured using Cell Proliferation ELISA BrdU colorimetric kit (Roche, Basel, Switzerland). Briefly, isolated T-cells were seeded into 96-well flat bottom plates at the density of 10^5^ cells per well in the medium described above, containing 2.5 µg/ml of TA-AgNPs or TA-AuNPs with or without 5 µg/ml concanavalin A (Sigma-Aldrich). After 48 h of incubation bromodeoxyuridine (BrdU) was added to a final concentration of 100 µM, and cells were incubated for additional 24 h. Next, the cells were fixed and incorporated BrdU was detected according to the manufacturer′s protocol.

### Statistical Methods

Data are presented as the mean ± standard error of the mean (S.E.M.) from at least three independent experiments. Data were analyzed using a two-tailed paired Student′s *t*-test (normal distribution) or non-parametric Kruskal–Wallis and Wilcoxon tests were applied using Biostat 2009 software. In every analysis, values of *p* ≤ 0.05 were considered significant.

## Results

### NPs Characterization

Before biological tests, AuNPs and AgNPs were precisely characterized. Briefly, the shape and size of metallic core of NPs were determined with STEM technique, the hydrodynamic size of NPs (the size of a metallic core along with substances present on the NPs surface) was measured with DLS technique and the colloidal stability of NPs was studied with DLS, UV–vis spectroscopy, and Zeta potential measurements.

**Figure 1:**
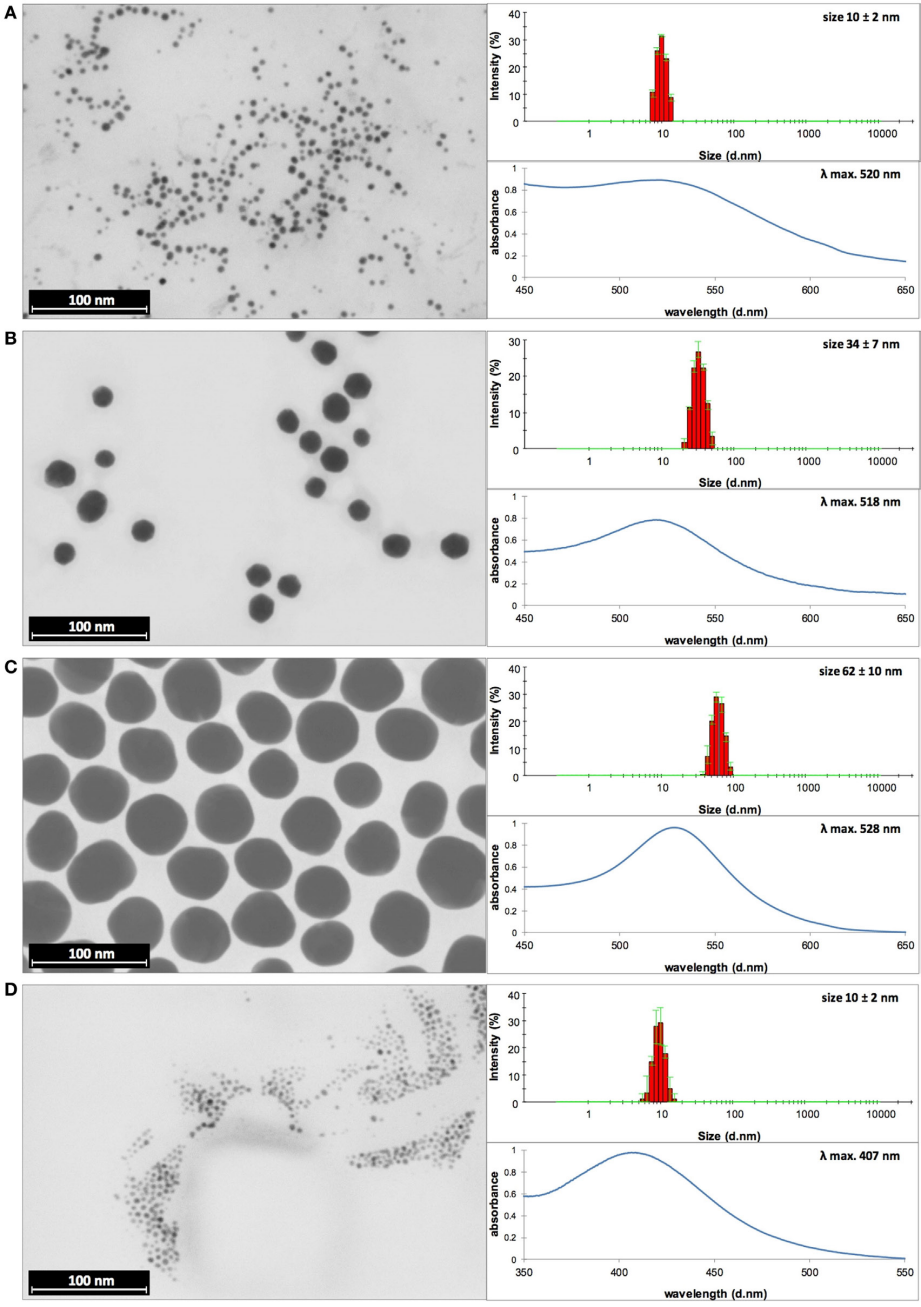

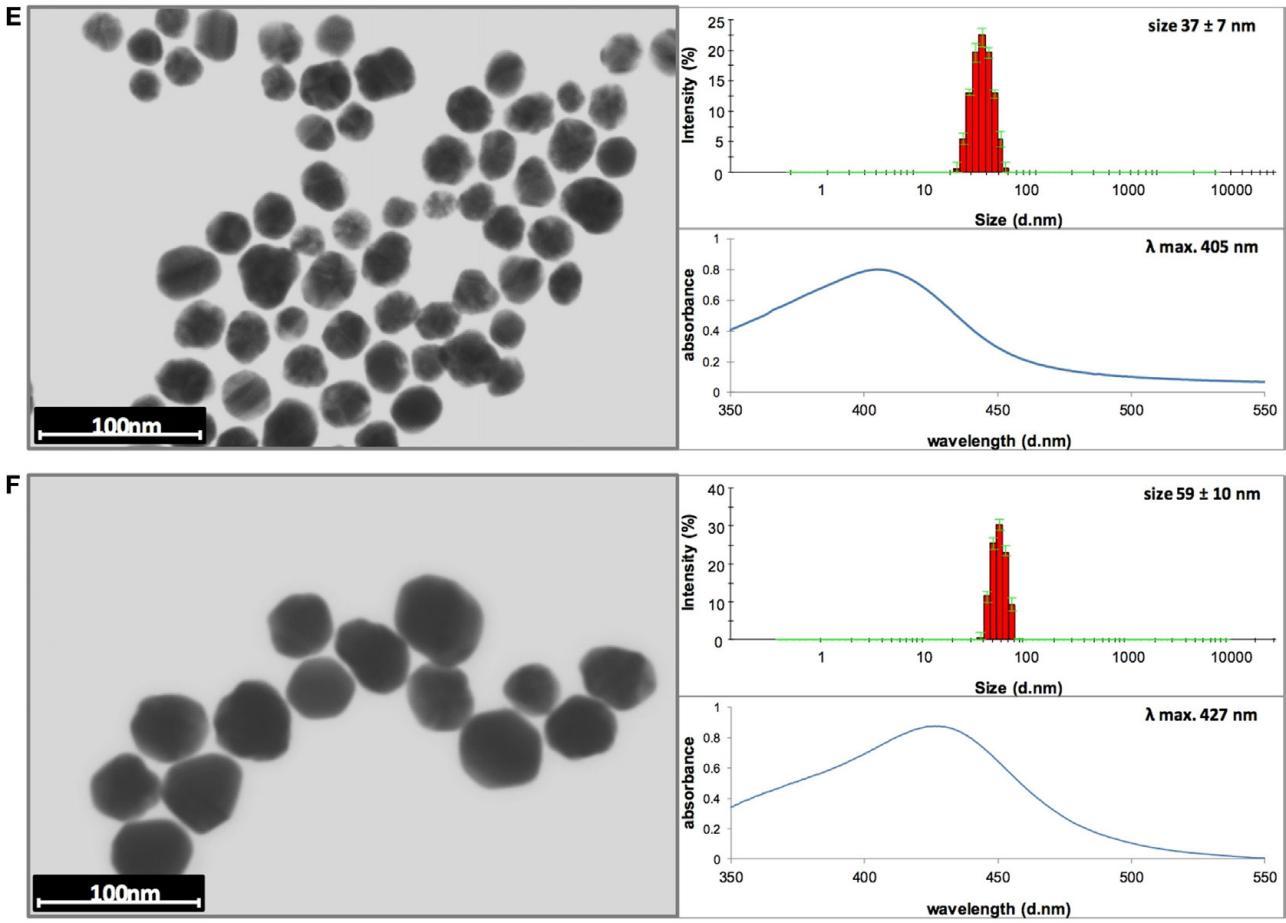
Dynamic light scattering (DLS) histograms, UV–vis, and scanning transmission electron microscopy images with size distribution histograms of AuNPs sized **(A)** 10 nm, **(B)** 34 nm, and **(C)** 62 nm, and AgNPs sized **(D)** 10 nm, **(E)** 37 nm, and **(F)** 59 nm.

The STEM images along with the DLS size distribution histograms and UV–vis spectra of AuNPs and AgNPs are presented in Figures 1A–F (AuNPss and AgNPs, respectively). The size of a metallic core of AuNPs is 5 ± 1 nm (Figure 1A), 24 ± 3 nm (Figure 1B), and 58 ± 7 nm (Figure 1C), and the size of a metallic core of AgNPs: 6 ± 2 nm (Figure 1D), 27 ± 7 nm (Figure 1E), and 45 ± 8 nm (Figure 1F). The hydrodynamic size of NPs is: 10 ± 2 nm (Figure 1A), 34 ± 7 nm (Figure 1B), 62 ± 10 nm (Figure 1C), 10 ± 2 nm (Figure 1D), 37 ± 7 nm (Figure 1E), and 59 ± 10 nm (Figure 1F). Any other peaks from agglomerates or aggregates of NPs were not detected in all cases, which confirm the monodispersity and colloidal stability of all samples. The differences between the hydrodynamic size of NPs measured in DLS technique and the size of metallic core measured in STEM technique correspond to the shell of stabilizers adsorbed on NPs surface. The shell of stabilizers adsorbed on NPs surface consist of complexes of tannic acid and sodium citrate (25), which are involved in the synthesis of NPs and its further stabilization in a colloidal solution. The absorption peaks maxima recorded by UV–vis spectroscopy were located at wavelengths characteristic for AuNPs at: 520 nm, 518 nm, and 528 nm (samples A, B, and C, respectively) and for AgNPs at: 407 nm, 405 nm, and 427 nm (samples D, E, and F, respectively). The negative values of Zeta potential confirmed high storage stability of all colloids (−26 ± 6 mV, −32 ± 1 mV, −51 ± 5 mV, −31 ± 7 mV, −58 ± 2 mV, and −56 ± 2 mV for sample A, B, C, D, E, and F, respectively). DLS results along with the UV–vis spectra and Zeta potential measurements confirm high stability of all investigated colloids. The overall results of AuNPs and AgNPs characterization are shown in Table 3.

**Table 3:** The overall results of AuNPs and AgNPs characterization.

Sample	STEM size (nm)	DLS size (nm)	UV–vis λ_max_ (nm)	Zeta potential (mV)
**AuNPs**				
A	5 ± 1	10 ± 2	520	−26 ± 6
B	24 ± 3	34 ± 7	518	−32 ± 1
C	58 ± 7	62 ± 10	528	−51 ± 5
**AgNPs**				
D	6 ± 2	10 ± 2	407	−31 ± 7
E	27 ± 7	37 ± 7	405	−58 ± 2
F	45 ± 8	59 ± 10	427	−56 ± 2

### Toxicity of NPs Is Size, Concentration, and Metal Dependent

**Figure 2:**
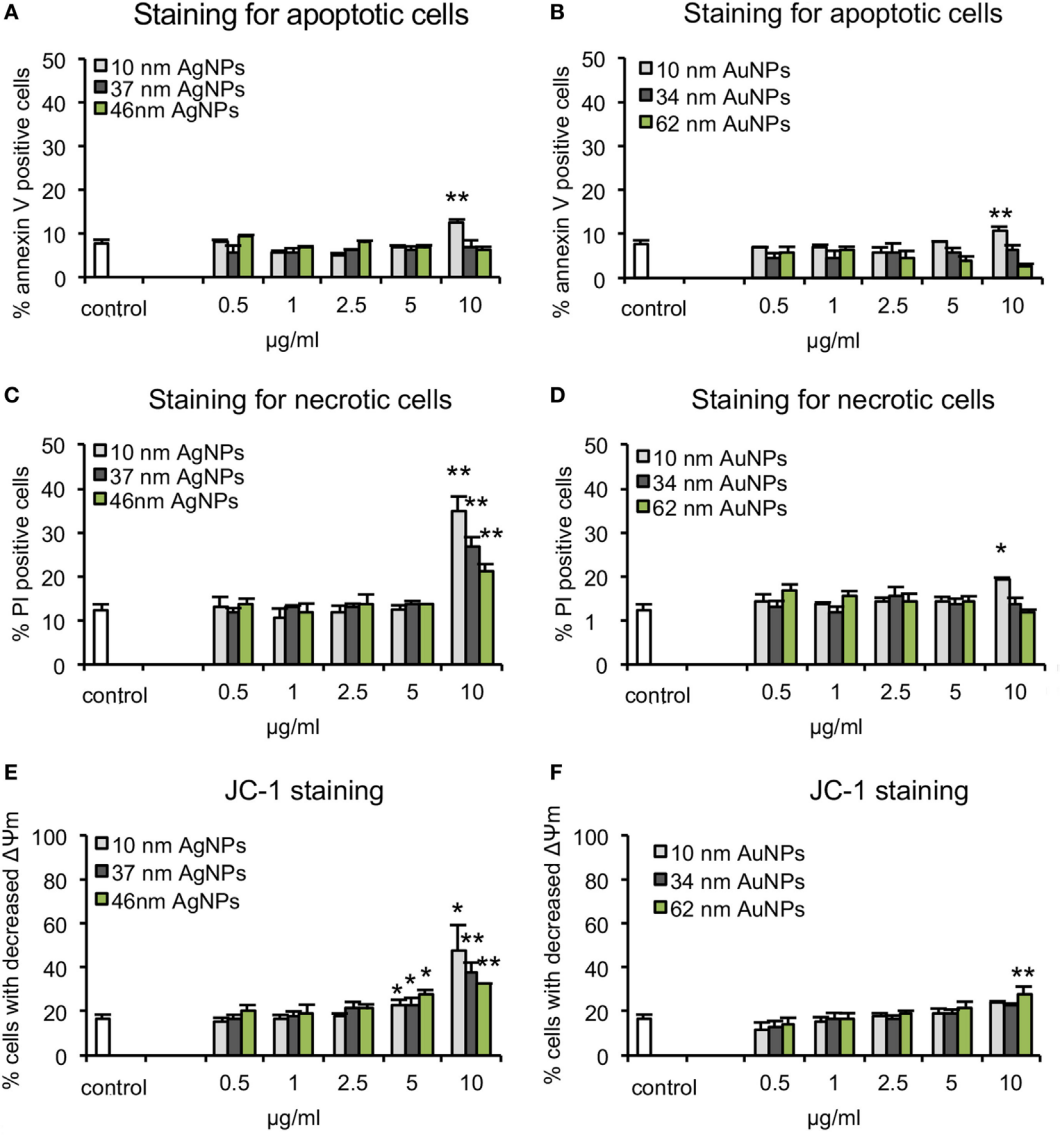
Cytotoxicity assays in JAWSII cell line at 24 h of exposure to AgNPs sized 10 nm, 37 nm, and 59 nm, and AuNPs sized 10 nm, 34 nm, and 62 nm at 0.5–10 µg/ml. **(A,B)** Percentage of annexin V-positive (apoptotic) cells. **(C,D)** Percentage of PI positive (necrotic) cells. **(E,F)** Decrease in mitochondrial potential expressed as the percentage of cells with decreased mitochondrial potential Each bar represents the mean from five experiments (*N* = 5) ± S.E.M., *Significant differences with *p* ≤ 0.05, **p ≤ 0.01.

The apoptosis/necrosis assay at 24 h of exposure to NPs showed that toxicity of NPs was size and concentration dependent but also metal dependent. We found that TA-AgNPs are more toxic than TA-AuNPs, with more necrotic than apoptotic cells at ≥5 µg/ml (Figure 2). Only the highest concentrations of the smallest TA-AgNPs and TA-AuNPs caused significant apoptosis (*p* ≤ 0.01) (Figures 2A,B). The significant increase of the necrotic cells was observed at 10 µg/ml for all tested TA-AgNPs (35.05 ± 3% for 10 nm, 26.64 ± 2.26% for 37 nm, and 20.88 ± 1.9% for 59 nm) (*p* ≤ 0.05) (Figure 2C). On the other hand, the significant increase in the percentage of necrotic cells for TA-AuNPs treatments was observed only for 10 nm at the 10 µg/ml (51.8 ± 3.09% in comparison to 39.39 ± 2.26% for control) (*p* = 0.034) (Figure 2D).

The mitochondrial potential after 24 h of treatment with TA-AgNPs or TA-AuNPs was determined using the JC-1 assay (Figures 2E,F). The decrease of mitochondrial potential was observed for all tested TA-AgNPs at ≥5 µg/ml and only for 62 nm TA-AuNPs at 10 µg/ml (*p* ≤ 0.05). Again, the strongest toxic effect was observed for 10 nm TA-AgNPs treatment—at 10 µg/ml, we observed 48.19 ± 11.08% cells with decreased mitochondrial potential in comparison to 37.32 ± 4.9% for 37 nm TA-AgNPs and 32.43 ± 0.29% for 59 nm TA-AgNPs (Figures 2E,F). These data suggest mitochondrial potential disruption and necrosis rather than apoptosis as the main toxic effects of tested NPs.

Toxicity tests in endothelial cells from mouse peripheral lymph nodes HECa10 showed results similar to those obtained in JAWS II, indicating metal- and size-dependent toxicity. We found 10 nm TA-AgNPs already toxic at 2.5 µg/ml, while for other NPs, toxicity started from 5 µg/ml (10 nm TA-AuNPs) or 10 µg/ml (all remaining NPs) (Table S1 in Supplementary Material).

### Uptake of NPs and Impact on Cellular Ultrastructure

**Figure 3:**
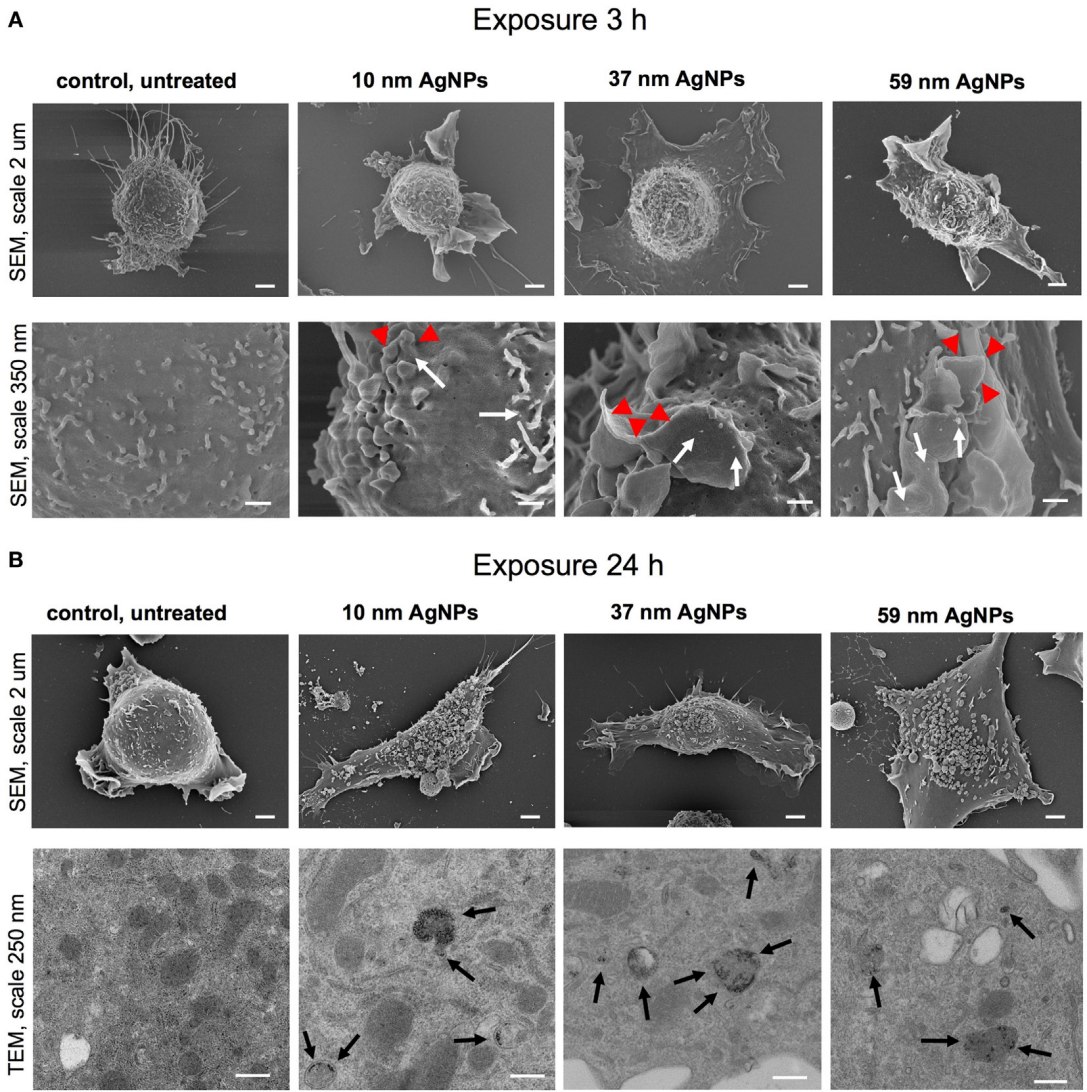
Scanning electron microscopy (SEM) and transmission electron microscopy (TEM) images of JAWS II cell line after 3 **(A)** and 24 h **(B)** of incubation with 5 µg/ml 10, 37, and 59 nm TA-AgNPs. White arrows indicate deposition of NPs, red arrowheads indicate lamellipodia, while black arrows point to intracellular deposits of AgNPs. Bars 2 μm, 250 nm and 350 nm are indicated.

**Figure 4:**
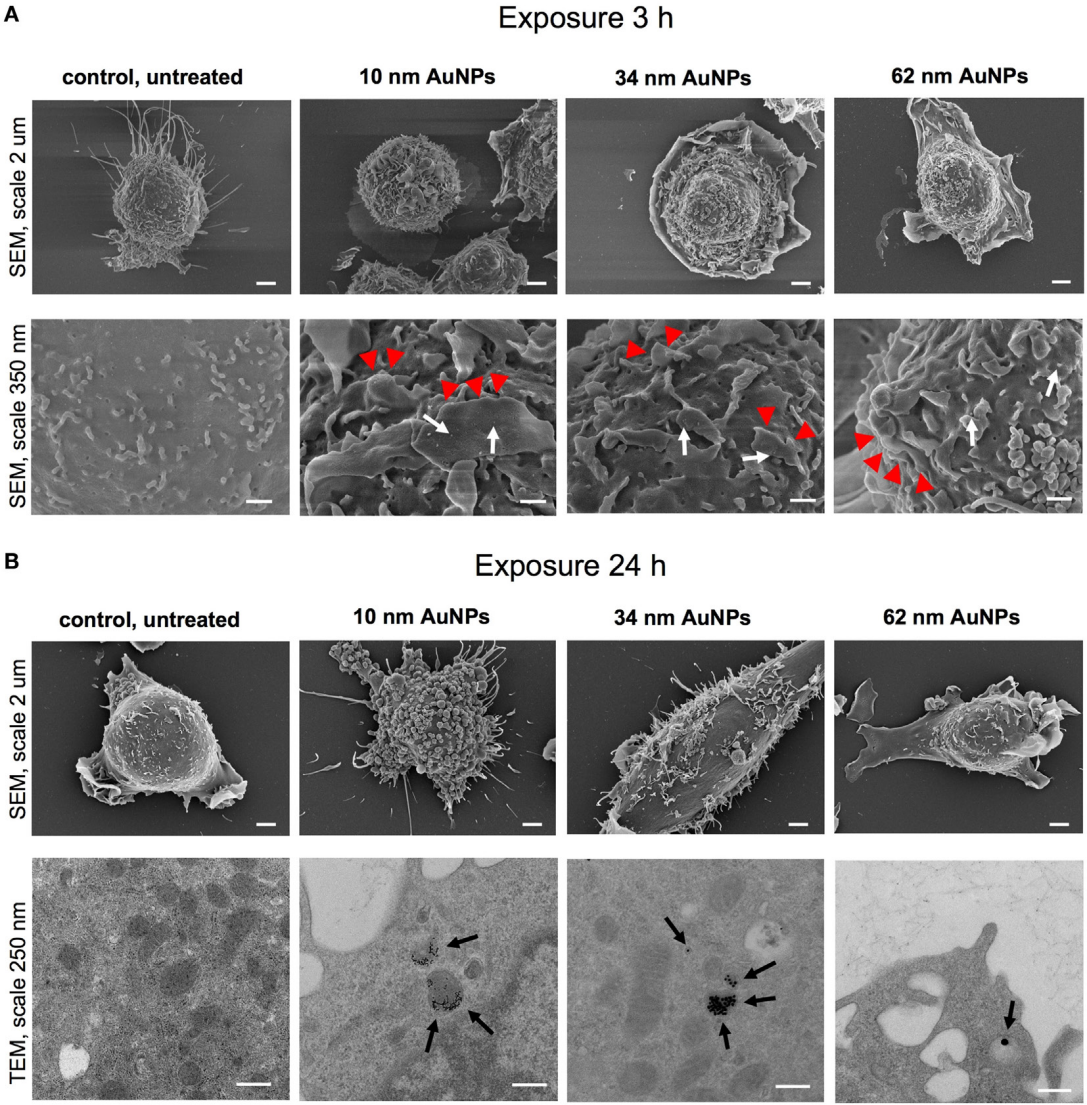
Scanning electron microscopy (SEM) and transmission electron microscopy (TEM) images of JAWS II cell line after 3 **(A)** and 24 h **(B)** of incubation with 5 µg/ml 10, 34, and 62 TA-AuNPs. White arrows indicate deposition of NPs, red arrowheads indicate lamellipodia, while black arrows point to intracellular deposits of AuNPs. Bars 2 μm, 250 nm and 350 nm are indicated.

To assess the influence of NPs upon morphology and ultrastructure of DCs, we used SEM (Figures 3 and 4). After 3 h of treatment with all tested NPs at 5 µg/ml, DCs showed an activated morphology (Figures 3A and 4A)—the cells became less rounded and more adherent in comparison to the untreated control cells (Figures 3A and 4A). Surface ultrastructure of cells exposed to NPs showed numerous microvilli of various length and lamellipodia with funnel configuration decorated with single or small groups of NPs (Figures 3A and 4A). These structures indicate phagocytosis and endocytosis as the main mechanism of NPs uptake. The activated morphology of DCs is retained at 24 h of exposure to NPs (Figures 3B and 4B).

To investigate intracellular localization of NPs in exposed cells, we employed TEM (Figures 3B and 4B). The 24 h exposure of JAWS II cells to NPs at the concentration of 2.5 µg/ml led to accumulation of NPs mainly within vesicles filled with an electron dense content. We found that intracellular sizes of all tested AgNPs were below the synthesis dimensions (Figure 3B) in contrast to TA-AuNPs with the unchanged sizes (Figure 4B). TA-AgNPs of 10 nm and 37 nm as well as TA-AuNPs of 10 nm and 34 nm were localized as attached to inner vesicles membrane (Figures 3B and 4B, respectively) whereas 59 nm TA-AgNPs were found as small groups of particles dispersed in vesicles (Figure 3B). Single 62 nm TA-AuNPs were thoroughly distributed in the vesicles content (Figure 4B).

**Figure 5:**
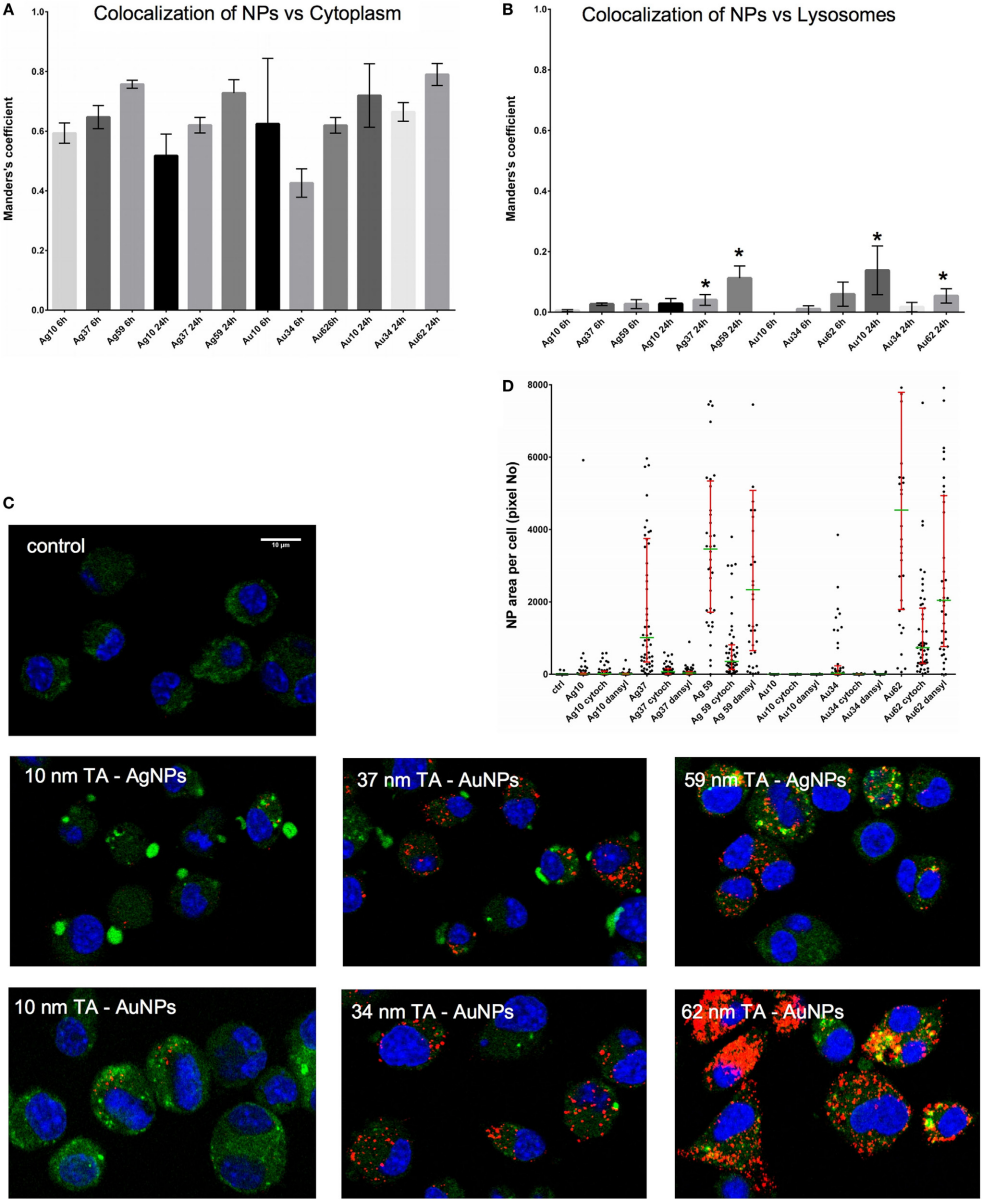
Intracellular localization of TA-Ag/AuNPs. The Manders’ coefficients for co-localization of TA-Ag/AuNPs and cytoplasm **(A)** or lysosomes **(B)** in JAWS II cell culture exposed to 10 nm, 37 nm, 59 nm TA-AgNPs and 10 nm, 34 nm, 62 nm TA-AuNPs for 24 h at 2.5 µg/ml. *Significant differences with *p* ≤ 0.05. **(C)** Representative images for lysosomes (green), nanoparticles (NPs) (red) and nuclei (blue) in cells exposed to NPs, as described above. **(D)** NPs content in cells subjected to pretreatment with 10 µg/ml monodansyl cadeverine and 5 µg/ml cytochalasin D, and then to incubation with TA-Ag/AuNPs at 2.5 µg/ml for 6 h.

To test if NPs are degraded in lysosomes, we stained cells with LysoTracker (Figure 5). The analyses showed that co-localization of TA-Ag/AuNPs with lysosomes was lower in comparison to localization in non-acidic compartments both at 6 and 24 h of incubation (Figures 5A,B). The highest co-localization with lysosomes was observed for 37 nm and 59 nm TA-AgNPs, 10 nm and 62 nm TA-AuNPs at 24 h (Figure 5B). Figure 5C shows localization of TA-Ag/AuNPs in JAWS stained for lysosomes. The NPs are visualized in red by the light reflected from their surface. Therefore, the higher surface to volume ratio, the higher signal (Figure 5C).

To further understand the mechanism of NPs uptake, we used pharmacological inhibitors of clathrin-mediated endocytosis (MDC), caveolae/lipid raft-mediated endocytosis (genistein), colchicine (pinocytosis), fluid-phase endocytosis (wortmannin), phagocytosis, and macropinocytosis (CChD). Of all used inhibitors, only MDC and CChD significantly inhibited uptake of all NPs at 6 hours of incubation (*p* ≤ 0.05) (Figure 5D).

### Effect of NPs on Expression of Surface Activation Markers

**Figure 6:**
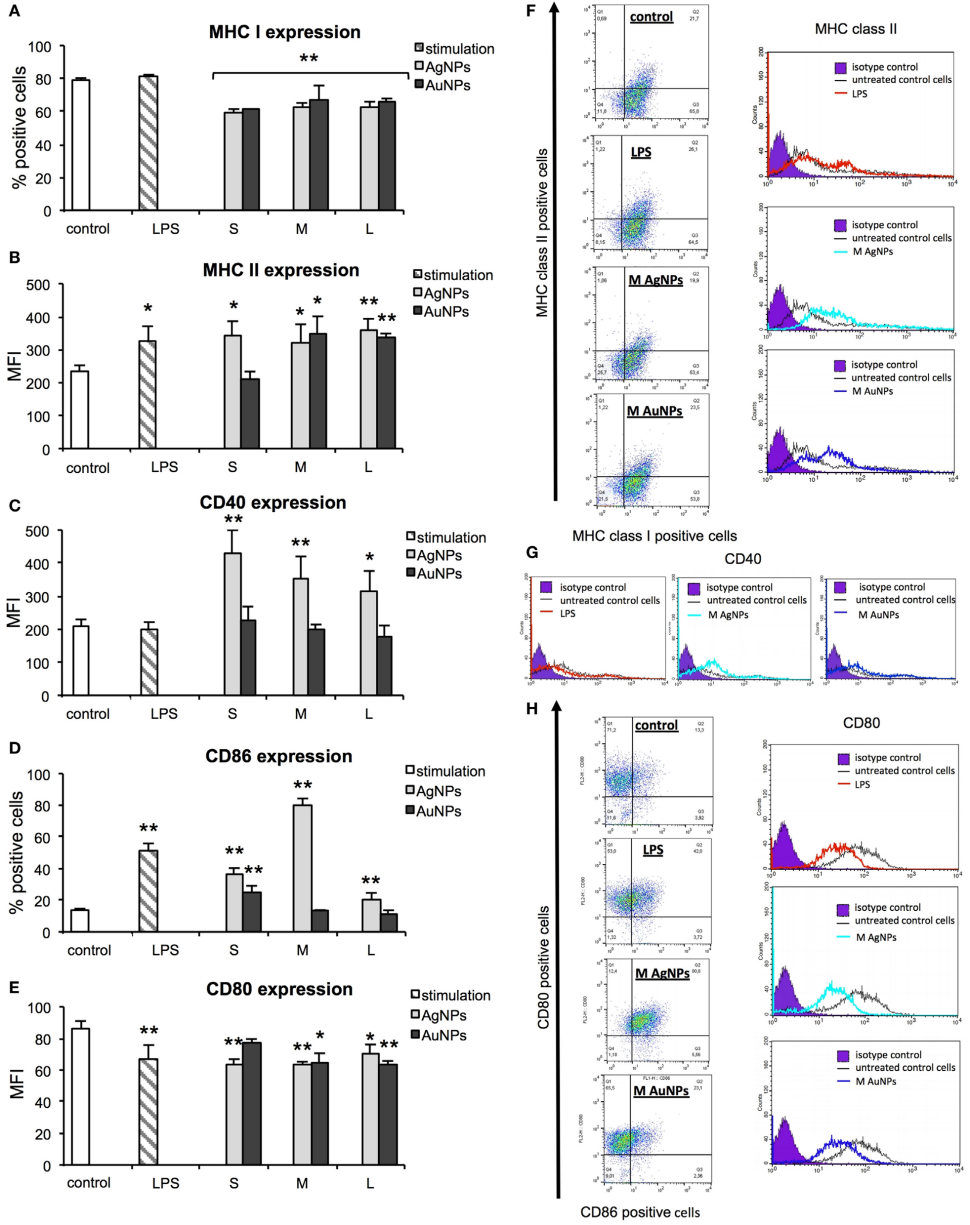
Effect of nanoparticles (NPs) on expression of surface activation markers. MHC class I **(A)**, MHC class II **(B)**, CD40 **(C)**, CD86 **(D)**, and CD80 **(E)** from 5 experiments (*N* = 5) ± S.E.M. **(F)** Representative dot plots (left panel) for MHC I and MHC II expression and representative histograms for MHC II expression. **(G)** Representative histograms for CD40 expression. **(H)** Representative dot plots for CD80 and CD86 expression (left panel) and representative histograms for CD86 expression (right panel). *Significant differences with *p* ≤ 0.05, **p ≤ 0.01.

**Figure 7:**
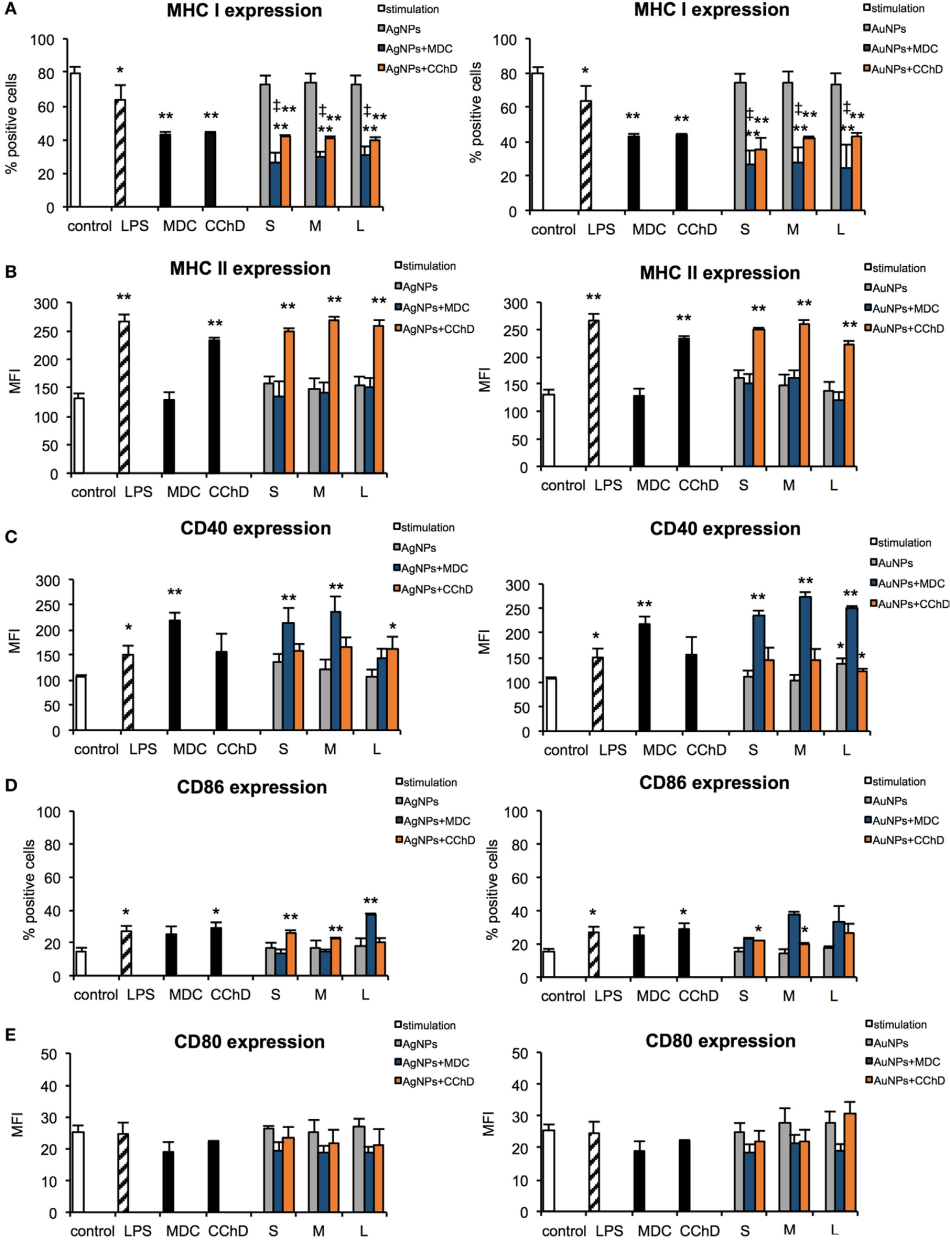
Effect of nanoparticles (NPs) on expression of surface activation markers in the presence of endocytosis and phagocytosis inhibitors. MHC class I **(A)**, MHC class II **(B)**, CD40 **(C)**, CD86 **(D)**, and CD80 **(E)** expression on the JAWS II cells after 6 h exposure to 2.5 µg/ml 10 nm (S), 37 nm (M), 59 nm (L) TA-AgNPs and 10 nm (S), 34 nm (M), 62 nm (L) TA-AuNPs with or without pretreatment with 10 µg/ml monodansyl cadeverine (MDC), and 5 µg/ml cytochalasin D (CChD). Each bar represents the mean from three experiments (*N* = 3) ± S.E.M. *Significant differences with *p* ≤ 0.05, **p ≤ 0.01 in comparison to untreated control, ‡Significant differences with *p* ≤ 0.05 in comparison to inhibitor treatment.

During activation and maturation, DCs change their morphology and up-regulate surface activation markers. To check the influence of NPs on maturation of DCs, we measured expression levels of MHC class I/II and CD40, CD80, and CD86 co-stimulatory molecules after 24 h treatment with NPs at the non-toxic dose of 2.5 µg/ml (Figure 6), but also at 6 h after treatment with NPs and inhibitors of endocytosis and phagocytosis—MDC and CChD (Figure 7). Lipopolysaccharide was used to stimulate DCs maturation in the positive control. For JAWS II, MHC I and CD80 expression is present on all cells, while MHC II, CD40, and CD86 are detected only for the percentage of cell culture. However, these cells show different characteristics of activation markers in response to NPs modified with tannic acid. While for MHC I and CD86, activation was observed as an increased or decreased percentage of cells expressing a particular marker, for CD40, CD80, and MHC II, activation was showed as changes in the mean fluorescence intensity (MFI). All tested NPs significantly decreased the percentage of MHC I positive cells (*p* ≤ 0.01) (Figure 6A). We observed 59.16 ± 2.24% and 61.49 ± 8.9% MHC I positive cells for small (S, 10 nm and 10 nm) TA-AgNPs and TA-AuNPs, 62.08 ± 3.06% and 66.84 ± 2.39% for medium (M, 37 nm and 34 nm) TA-AgNPs and TA-AuNPs, 62.13 ± 3.73% and 65.46 ± 3.75% for large (L, 59 nm and 62 nm) TA-AgNPs and TA-AuNPs, respectively, in comparison to control untreated cells (79.1 ± 1.19%) and LPS-stimulated positive control (89.04 ± 1.81%) (*p* = 0.002) (Figure 6A). MDC and CChD significantly reduced MHC I expression in control JAWS II cells at 6 h (*p* ≤ 0.01) (Figure 7A), which can be contributed to the individual sensitivity of this cell line to phagocytosis and endocytosis inhibitors. However, only treatment with MDC led to further significant decrease in MHC I expression in cells treated with all sizes of TA-Ag/AuNPs in comparison to MDC-treated control cells at 6 h (*p* ≤ 0.05) (Figure 7A). In contrast, all NPs except for 10 nm TA-AuNPs significantly increased MHC II expression, at the level similar to LPS stimulation (339.36 ± 4.68 MFI), in comparison to untreated control (233.93 ± 18.15 MFI) (*p* ≤ 0.05) (Figure 6B). Significant increase of MHC II expression was also observed for cells treated with CChD, both control and NPs-treated at 6 h (*p* ≤ 0.05) (Figure 7B).

Analysis of CD40 revealed that DCs exposed to TA-AgNPs increase expression of this marker (Figure 5C). Stimulation with 10 nm TA-AgNPs led to a two-fold increase of CD40 expression in comparison to untreated control cells (431.69 ± 67.81 MFI and 209.36 ± 20.18 MFI, respectively) (*p* ≤ 0.01). Exposure to 37 nm and 59 nm TA-AgNPs increased CD40 expression to 354.39 ± 65.83 MFI (*p* ≤ 0.01) and 317.23 ± 58.83 MFI (*p* ≤ 0.05), respectively. No significant changes were observed upon TA-AuNPs or LPS treatment (*p* ≥ 0.05) (Figure 6C).

Interestingly, NPs-treated cells subjected to MDC significantly up-regulated CD40 expression in comparison to control and MDC-treated control (*p* ≤ 0.05), except for 59 nm TA-AgNPs (Figure 7C). CChD did not influence CD40 expression in NPs-treated cells (Figure 7C).

Up-regulation of CD86 expression was found during LPS, TA-AgNPs, and 10 nm TA-AuNPs treatment (Figure 6D). Treatment with 10 nm TA-AgNPs or TA-AuNPs led to a three- and two-fold increase in CD86 expression (36.51 ± 3.83% and 25.21 ± 3.91%, respectively) in comparison to control (13.78 ± 0.96%). Interestingly, treatment with 37 nm TA-AgNPs elevated CD86 expression five-fold (80.46 ± 3.84%) in comparison to untreated control cells (13.78 ± 0.96%) (*p* = 0.0000). No significant influence upon CD86 expression was found for cells treated with MDC, both control and NPs-treated at 6 h (*p* > 0.05) (Figure 7D).

A significant decrease in CD80 expression levels was observed upon LPS treatment (66.42 ± 9.37 MFI) as well as for 10 nm (62.94 ± 3.89 MFI), 37 nm (63.42 ± 1.79 MFI), and 59 nm (69.99 ± 6.12) TA-AgNPs treatment when compared with control cells (86.19 ± 4.79 MFI) (*p* ≤ 0.01) (Figure 6E). Significantly decreased expression of CD80 was also observed during exposure to 34 and 62 TA-AuNPs (64.08 ± 6.49 MFI and 63.48 ± 2.25 MFI, respectively) (*p* ≤ 0.01) (Figure 6E). No significant influence upon CD80 expression was found for cells treated with MDC, both control and NPs-treated at 6 h (*p* > 0.05) (Figure 7E).

Programmed death-ligand 1 (PD-L1) has been postulated to play a crucial role in suppressing lymphocyte activation (26). Here, we tested if TA-NPs treatment has any influence upon PD-L1 expression in JAWS II cells, but we found no significant differences (data not shown).

To check for relevance with *in vivo* conditions, we performed follow-up experiments with BMDCs cultures obtained from C57BL/6 mice. All sizes of TA-AgNPs significantly decreased percentages of MHC I positive BMDCs (*p* ≤ 0.05), while this tendency was insignificant for TA-AuNPs treated cells (*p* > 0.05) (Figure S1A in Supplementary Material). BMDCs treated with all sizes of TA-AgNPs/AuNPs significantly increased MHC II expression, at the level similar to LPS stimulation in comparison to untreated control (*p* ≤ 0.05) (Figure S1B in Supplementary Material). As for JAWS II cells, BMDCs exposed to TA-AgNPs significantly increased expression of CD40 (*p* ≤ 0.05) (Figure S1C in Supplementary Material). A decrease in CD80 expression levels was observed upon LPS treatment and treatment with all sizes of TA-AgNPs/AuNPs (*p* ≤ 0.05) (Figure S1D in Supplementary Material) except for 10 nm and 37 nm TA-AgNPs, due to a high distribution of individual results. For CD86 expression, we did not observe any significant differences in the expression patterns after exposure to TA-AgNPs/AuNPs (*p* > 0.05) (Figure S1D in Supplementary Material).

### NPs Stimulate Uptake of Viral Antigens

**Figure 8:**
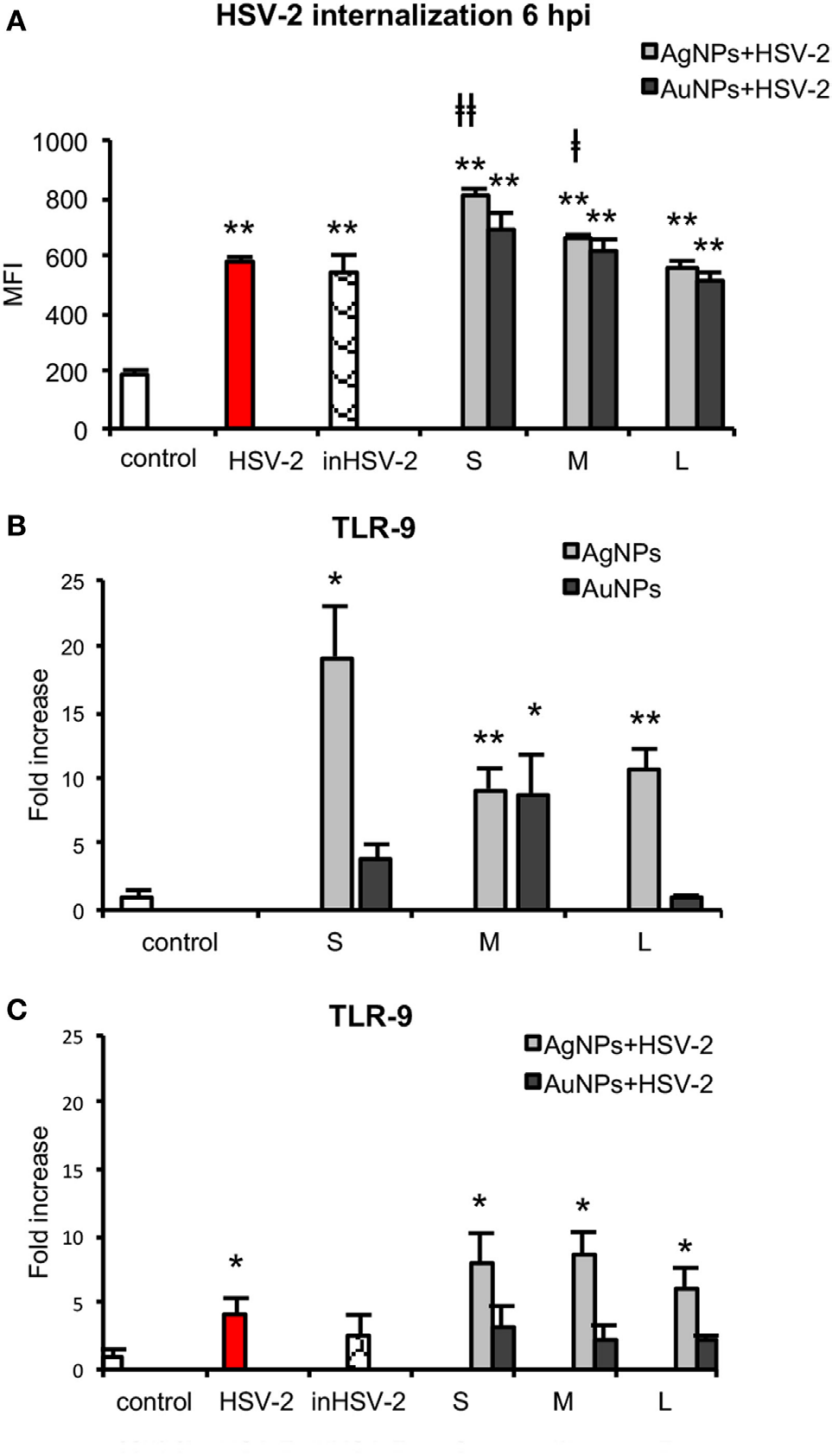
Nanoparticles (NPs) stimulate uptake of viral antigens and expression of TLR9. **(A)** Presence of HSV-2 antigens in JAWS II cells after 6 h exposure to HSV-2, inactivated HSV-2, or NPs-treated HSV-2. TLR 9 expression in NPs- and HSV-NPs-treated JAWS II cells. The cells were exposed to 2.5 µg/ml of **(B)** TA-Ag/AuNP, **(C)** TA-AgNPs or TA-AuNPs-treated HSV-2. Each bar represents the mean from three experiments (*N* = 3) ± S.E.M. *Significant differences with *p* ≤ 0.05, **p ≤ 0.01 in relation to untreated control, ǂ*p* ≤ 0.05, and ǂǂ*p* ≤ 0.01 calculated in relation to the HSV-2 infected control.

In our previous work, we showed that 1 h pre-incubation of HSV-2 inoculum with 2.5 µg/ml TA-NPs resulted in complete inhibition of infection *in vitro* (15). Here, we examined if TA-NPs-treated HSV-2 (HSV) can induce DCs maturation. First, we studied the level of TA-NPs-treated HSV-2 (NPs-HSV) uptake by JAWS II cells in comparison to non-treated HSV-2 and heat inactivated HSV-2 (inHSV) (Figure 8A). The antigen uptake after 6 h post infection (p. i.) for HSV and inHSV was observed at the similar level (578.84 ± 15.67 MFI and 541.06 ± 60.78 MFI, respectively). A six-hour incubation of JAWS II with 2.5 µg/ml TA-NPs-HSV resulted in a significant internalization of viral antigens in the presence of S and M AgNPs-HSV (*p* ≤ 0.05) in comparison to HSV-2 infected control. The highest observed antigen uptake was found for S TA-AgNPs-HSV (809.89 ± 21.79 MFI) (*p* = 0.0005) (Figure 8A). Blockage of the virus surface by L TA-AgNPs and all tested TA-AuNPs did not influence the uptake of viral antigens in comparison to infection with untreated HSV-2.

### TLR9 Expression

TLR9 is expressed within the endoplasmic compartment and recognizes CpG DNA motifs (27). Since we observed higher internalization of HSV-2 treated with NPs, we measured the level of TLR9 mRNA in the treated JAWS II cells. After exposure of DCs to TA-AgNPs, the level of TLR9 mRNA significantly increased inversely with their size (Figure 8B). Exposure to 10 nm TA-AgNPs induced 26.02 ± 3.82-fold higher expression of TLR9 mRNA in comparison to control cells (*p* = 0.046) (Figure 8B). The 37 nm and 59 nm TA-AgNPs caused 9.19 ± 1.53 and 10.54 ± 1.66-fold increase of TLR9 mRNA level in comparison to control, respectively (*p* ≤ 0.01). Only 37-nm-sized TA-AuNPs caused a significant increase in the TLR9 mRNA level (8.86 ± 2.9) (*p* = 0.0280). Infection of JAWS II cells led to 4.11 ± 1.23 higher level of TLR9 mRNA in comparison to uninfected control cells (*p* = 0.039) (Figure 8B). During exposure of JAWS II cells to HSV-2 treated with TA-AgNPs, we observed an increased synthesis of mRNA (*p* ≤ 0.05) (Figure 8C). None of TA-AuNPs-treated HSV-2 significantly induced the synthesis of TLR9 mRNA level in comparison to untreated control (*p* ≥ 0.05) (Figure 8C).

### NPs Treated HSV-2 Stimulate Maturation of DCs

**Figure 9:**
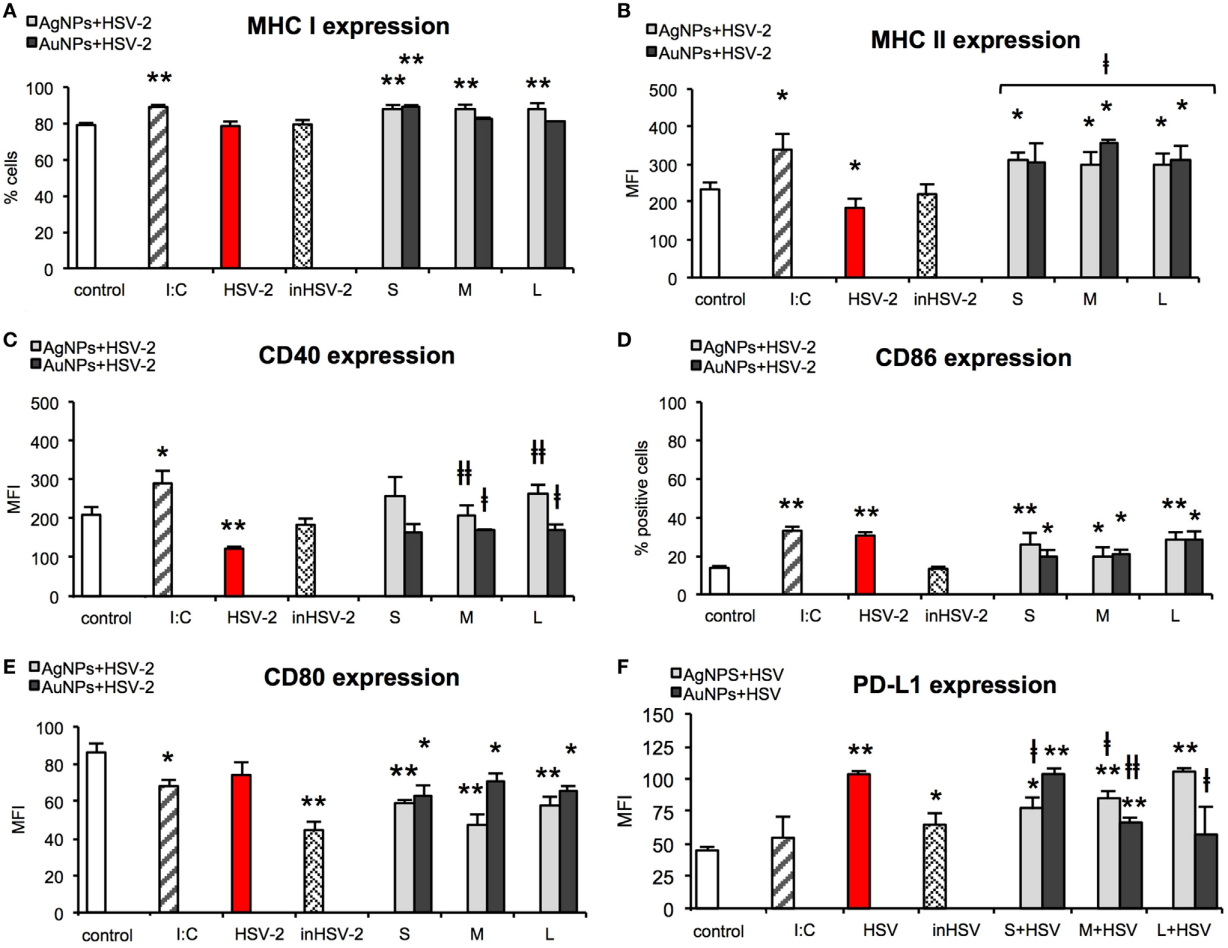
Nanoparticles (NPs)-treated HSV-2 stimulate maturation of dendritic cells (DCs). MHC class I **(A)**, MHC class II **(B)**, CD40 **(C)**, CD86 **(D)**, CD80 **(E)**, and PD-L1 **(F)** expression on the JAWS II cells after 24 h exposure to HSV-2 treated with 2.5 µg/ml of 10 nm (S), 37 nm (M), 59 nm (L) TA-AgNPs and 10 nm (S), 34 nm (M), 62 nm (L) TA-AuNPs. Each bar represents the mean from 5 experiments (*N* = 5) ± S.E.M. *Significant differences with *p* ≤ 0.05, **p ≤ 0.01 in relation to untreated control, ǂ*p* ≤ 0.05, and ǂǂ*p* ≤ 0.01 calculated in relation to the HSV-2 infected control.

Taking into account the fact that HSV-2 infection inhibits DCs maturation and thus results in a poor development of the specific immune response (28), we examined the impact of HSV-2 treated with NPs on the activation markers at 24 h post infection (p. i.). For all tested AgNPs-treated HSV-2, MHC class I expression was significantly higher in comparison to control cells and stayed at the level similar to that observed for positive poly (I:C) stimulated control (*p* ≤ 0.01) (Figure 9A). Following infection with TA-AuNPs-HSV-2, only 10 nm TA-AuNPs-HSV-2 significantly increased MHC I expression (*p* ≤ 0.01). HSV-2 infection significantly decreased MHC II expression in comparison to untreated control (184.63 ± 14.35% and 233.93 ± 14.15%, respectively) (*p* = 0.016) (Figure 9B). On the contrary, exposure to TA-AgNPs-treated HSV-2 resulted in a significant up-regulation of MHC II expression in comparison to untreated control and HSV-2 infected cells (*p* ≤ 0.05). Only exposure to M and L TA-AuNPs-treated HSV-2 caused a significant up-regulation of MHC II expression (354.47 ± 10.78 MFI and 313.98 ± 35.36 MFI, respectively) in comparison to untreated and HSV-2 infected cells (*p* ≤ 0.05).

HSV-2 infection also led to a significant decrease in CD40 expression (122.26 ± 5.41 MFI) in comparison to untreated control cells (209.36 ± 20.18 MFI) (*p* = 0.0075) (Figure 9C). However, cells exposed to HSV-2 treated with M and L TA-AgNPs showed significantly higher CD40 expression than HSV-2 infected cells (122.26 ± 5.41 MFI) (*p* ≤ 0.01). Also, cells exposed to HSV-2 treated with M and L TA-AuNPs showed significantly higher expression of CD40 (167.42 ± 4.17 MFI and 172.01 ± 12.69 MFI) in comparison to infected cells (122.26 ± 5.41 MFI) (*p* ≤ 0.05).

HSV infection of JAWS II cells resulted in a significant up-regulation of CD86 expression (30.07 ± 1.75%, *p* = 0.0000), as that observed for poly (I:C) stimulated control (33.06 ± 2.12%, *p* = 0.0000) in comparison to untreated control cells (13.78 ± 0.96%) (Figure 9D). All NPs-treated HSV-2 preparations significantly increased CD86 expression in comparison to untreated control cells (*p* ≤ 0.05). The lowest up-regulation of CD86 expression was observed for M TA-AgNPs and S/M AuNPs treated HSV-2 (20.2 ± 4.3%, 20.05 ± 3.03%, and 20.42 ± 2.8%, respectively) (*p* ≤ 0.05).

CD80 expression was significantly decreased upon poly (I:C) stimulation in comparison to untreated control (68.03 ± 3.42 MFI and 86.19 ± 4.79 MFI, respectively) (*p* = 0.0179) (Figure 9E). Inactivated HSV-2 was the strongest down-regulator of CD80 expression (44.38 ± 4.46) (*p* = 0.0015). All NPs-treated HSV-2 significantly downregulated expression of CD80. We observed stronger down-regulation of CD80 expression on the cells exposed to TA-AgNPs-HSV-2 than on the cells exposed to TA-AuNPs-HSV-2 preparations (*p* ≤ 0.01 and *p* ≤ 0.05, respectively) (Figure 9E).

Here, we checked if NPs-treated HSV-2 was able to influence the level of PD-L1 expression on JAWS II (Figure 9F). HSV-2 infection of JAWS II resulted in a significant up-regulation of PD-L1 (103.53 ± 2.36 MFI) in comparison to untreated control cells (44.68 ± 2.7 MFI) (*p* = 0.0000). We observed a size-dependent influence of TA-AgNPs-treated HSV-2 upon PD-L1 expression with PD-L1 expression increasing together with the size of AgNPs used for HSV-2 inactivation (*p* ≤ 0.01) (Figure 9F). Interestingly, treatment of HSV-2 with S/M TA-AgNPs led to PD-L1 expression which was lower than that this observed on HSV-2 infected DCs (*p* ≤ 0.05) (Figure 9F). For AuNPs-treated HSV-2, we observed a reversed correlation—PD-L1 expression decreased with the increasing size of AuNPs and the highest expression of PDL-1 was detected in S AuNPs-HSV-2-treated cells (Figure 9F). Furthermore, only M and L TA-AuNPs-treated HSV-2 preparations led to PD-L1 expression lower than that this observed for HSV-2 infection (*p* ≤ 0.05) (Figure 9F).

Polymyxin B had no significant influence upon expression of MHC I, MHC II, CD80, CD86 in DCs subjected to TA-NPs-treated HSV-2, except for CD40, expression of which was significantly down-regulated upon polymyxin B addition (Table S2 in Supplementary Material). Significant decrease in CD40 expression was observed for HSV-2 treated with TA-AgNPs, and 63 nm TA-AuNPs (*p* ≤ 0.05) (Table S2 in Supplementary Material).

### Effect of NPs and NPs Treated HSV-2 on Cytokine Secretion

**Figure 10:**
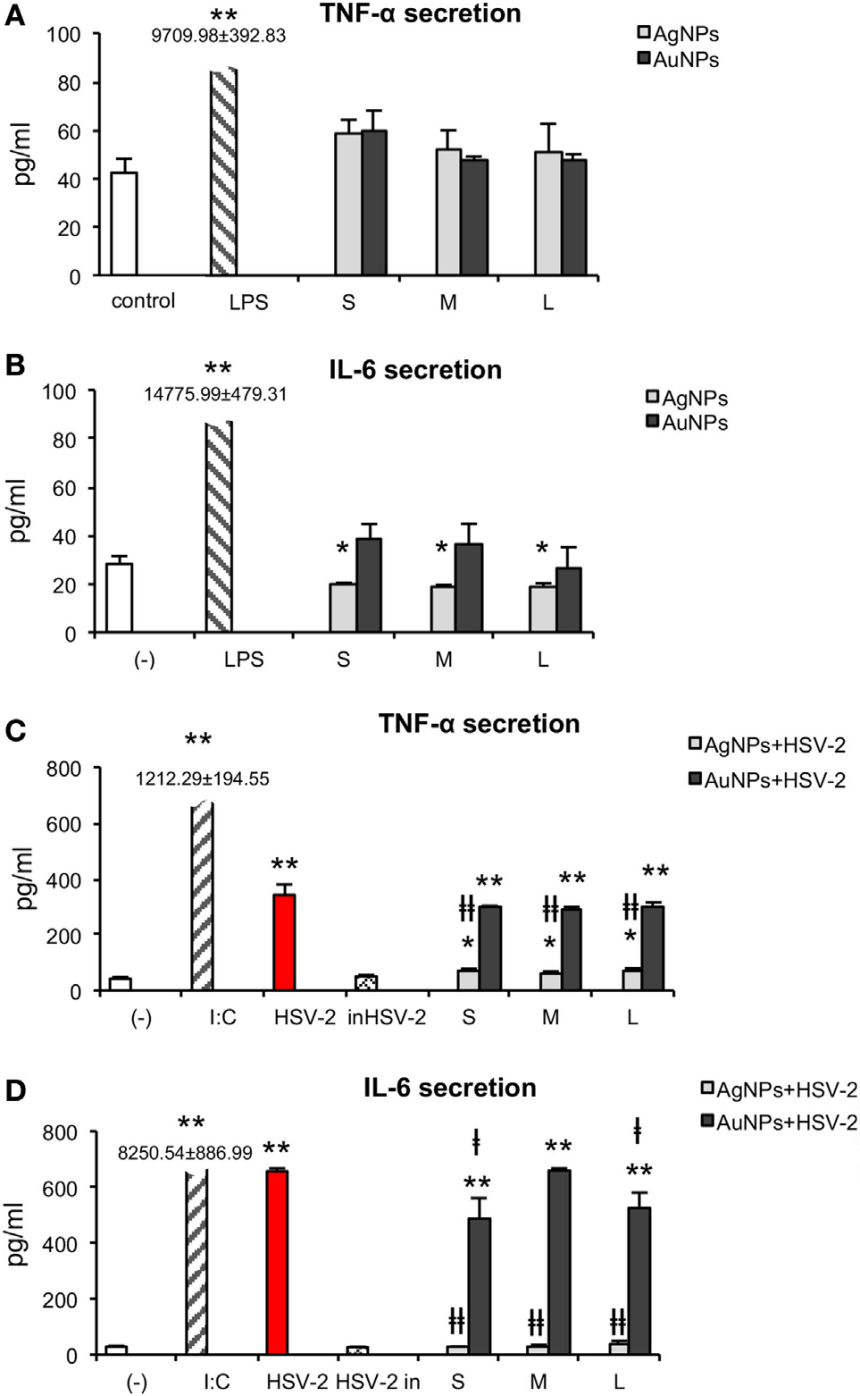
Effect of nanoparticles (NPs) and NPs-treated-HSV-2 on cytokine secretion. TNF-α **(A)** and IL-6 **(B)** production in JAWS II cells exposed to 2.5 µg/ml NPs for 24 h. **(C)** TNF-α and **(D)** IL-6 production in JAWS II cells exposed to NPs-treated HSV-2 for 24 h. Each bar represents the mean from 3 experiments (*N* = 3) ± S.E.M. *Significant differences with *p* ≤ 0.05, ** *p* ≤ 0.01 in relation to untreated control, ǂ*p* ≤ 0.05, and ǂǂ*p* ≤ 0.01 calculated in relation to the HSV-2 infected control.

During activation and maturation, DCs change the profile of produced cytokines. We measured secretion of IL-10, IL-17A, TNF-α, IFN-γ, IL-6, IL-4, and IL-2 by JAWS II subjected to HSV-2 treated with TA-Ag/AuNPs using the CBA. We were not able to detect IL-10, IL17A, IFN-γ, IL-4, or IL-2 in the supernatants from stimulated or control cells. Exposure to all NPs did not cause any significant change in TNF-α secretion in comparison to untreated control (Figure 10A) (*p* ≥ 0.05). IL-6 measurement showed a significant decrease upon treatment with S, M, and L TA-AgNPs (19.85 ± 0.55 pg/ml, 18.94 ± 0.69 pg/ml, and 19.2 ± 1 pg/ml, respectively) in comparison to untreated control cells (28.19 ± 3.22 pg/ml) (*p* ≤ 0.01) (Figure 10B).

HSV-2 infection of JAWS II significantly increased TNF-α production to 342.23 ± 37.59 pg/ml in comparison to 42.34 ± 5.89 pg/ml produced by untreated control cells (*p* = 0.0007) (Figure 10C). We did not observe any significant changes in TNF-α secretion upon stimulation with HSV-2 (*p* ≥ 0.05). Exposure to TA-AgNPs-treated HSV-2 caused significantly higher secretion of TNF-α, for all NPs sizes, in comparison to non-stimulated control (*p* ≤ 0.05), but it was significantly lower for all tested TA-AgNPs sizes in comparison to HSV-2 infected cells (71.01 ± 7.26 pg/ml for S, 64.18 ± 4.16 for M, and 74.32 ± 574 pg/ml for L TA- AgNPs-HSV-2) (*p* ≤ 0.01). On the other hand, stimulation with TA-AuNPs-treated HSV-2 significantly increased secretion of TNF-α at similar levels for all tested sizes (*p* ≤ 0.01). The observed increase was higher in TA-AuNPs-HSV-2 than in TA-AgNPs-HSV-2-treated cells. Interleukin 6 was produced during HSV-2 infection (656.45 ± 10.06 pg/ml), although at the level lower than in poly I:C stimulated control cells (8,250.42 ± 886.99 pg/ml) (*p* = 0.0000) (Figure 10D). In comparison to HSV-2 infected cells, all TA-AgNPs-HSV-2 preparations decreased IL-6 production (*p* ≤ 0.01) to the level observed in the unstimulated control (Figure 10D). TA-AuNPs-treated HSV-2 caused strong IL-6 production in comparison to untreated cells (*p* ≤ 0.01) (Figure 10D). Only S and L TA-AuNP-HSV-2 preparations significantly decreased production of IL-6 in comparison to HSV-2 infected cells (*p* ≤ 0.05) (Figure 10D).

### NPs Enhance the Ability of DCs to Activate Antigen-Specific T Cells *In Vitro*

**Figure 11:**
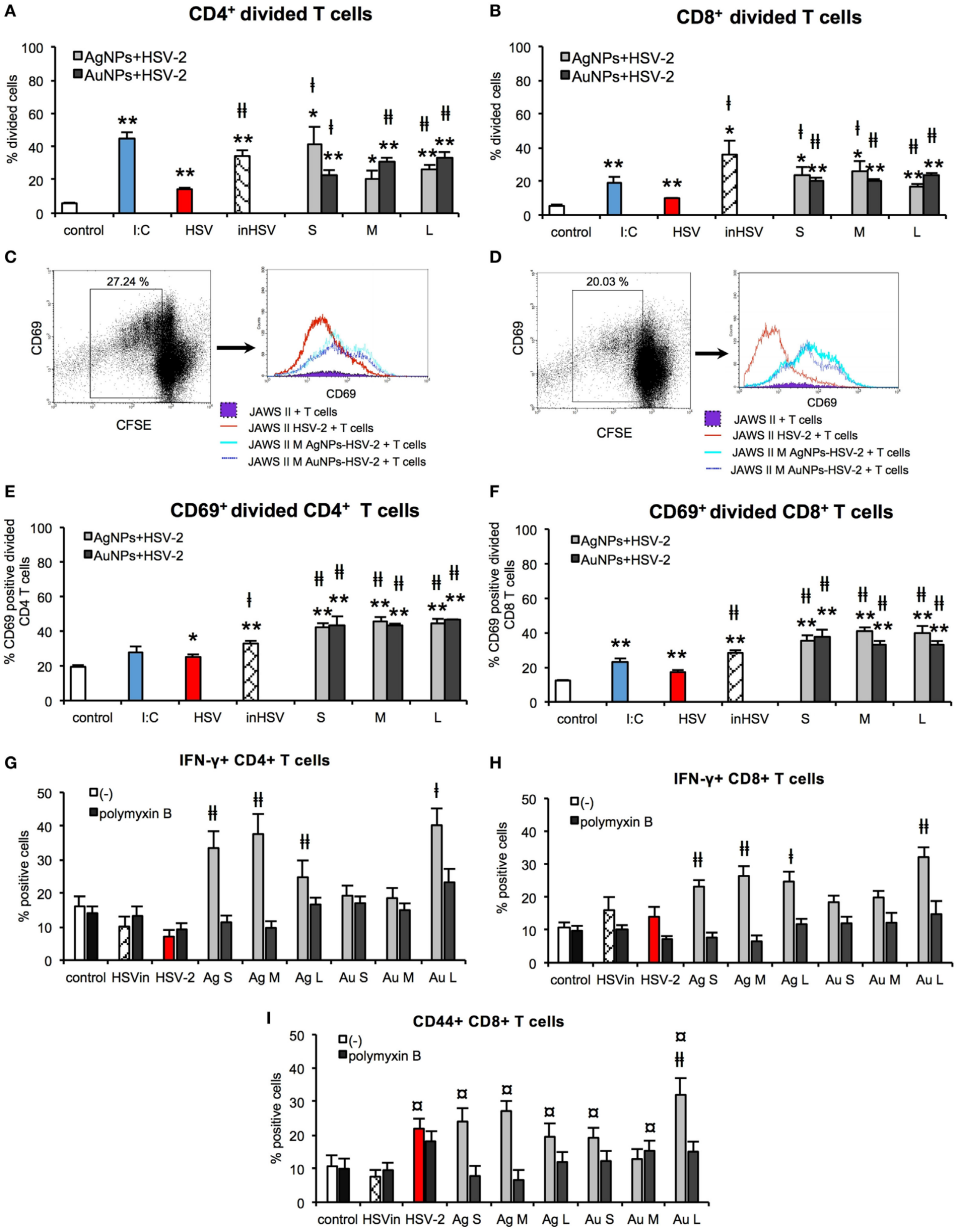
Nanoparticles (NPs) enhance the ability of dendritic cells (DCs) to activate T cells *in vitro*. Percentage of divided CD4+ **(A)** and CD8+ T cells **(B)**. Representative dot plots and histogram plots of CD69+ CD4+ **(C)** and CD69+ CD8+ **(D)** divided T cells. Percentage of CD69+ divided CD4+ **(E)** and CD69+ divided CD8+ T cells **(F)**. Percentage of IFN-γ+ CD4+ **(G)** and IFN-γ+ CD8+ T cells with or without 10 µg/ml polymyxin B **(H)**. Percentage of CD44+ CD8+ T cells with or without 10 µg/ml polymyxin B **(I)**. Each bar represents the mean from 3 experiments (*N* = 3) ± S.E.M. *Significant differences with *p* ≤ 0.05, **p ≤ 0.01 in relation to untreated control, ‡*p* ≤ 0.05, and ‡‡*p* ≤ 0.01 calculated in relation to the HSV-2 infected control. ¤*p* ≤ 0.01 calculated in relation to the inactivated HSV-2.

Development of adaptive immune response depends on the effective antigen presentation by APCs to naïve T cells. Here, we examined the ability of JAWS II exposed to TA-NPs-treated HSV-2 to activate splenic CD4^+^ and CD8^+^ T cells isolated from mice challenged with UV-inactivated HSV-2 (Figure 11). Treatment of JAWS II cells with untreated HSV-2 and heat inactivated HSV-2 resulted in induction of CD4^+^ and CD8^+^ T cells proliferation, with higher induction of CD4+ T cells than CD8+ T cells (*p* ≤ 0.01) (Figures 11A,B). Significant increase of CD4^+^ cells was observed if JAWS II were exposed to S and L TA-AgNPs-treated HSV-2 in comparison to HSV-2 infection (*p* ≤ 0.05) (Figure 11A). M TA-AgNPs-treated HSV-2 induced CD4+ T cells proliferation (*p* ≤ 0.05) (Figure 11A), albeit only in comparison to uninfected cells. JAWS II stimulated with all TA-AuNPs-treated HSV-2 induced significant proliferation of CD4^+^ cells in comparison to HSV-2 infected cells (*p* ≤ 0.05) (Figure 11A).

JAWS II cells exposed to all TA-NPs-HSV induced significantly higher proliferation of CD8^+^ cells in comparison to HSV-2 infected control (*p* ≤ 0.05) (Figure 11B). CD69 was used as a marker of early T cell activation in the population of divided cells. Dendritic cells subjected to all TA-NPs-HSV significantly induced expression of CD69 in divided CD4^+^ and CD8^+^ T cells in comparison to HSV-2 infected control cells (*p* ≤ 0.01) (Figures 11C–F). Furthermore, JAWS II cells exposed to all sizes of TA-AgNPs-HSV and L TA-AuNPs induced significantly higher percentages of IFN-γ CD4+ and CD8+ T cells in comparison to HSV-2 infected control or control cells treated with inactivated HSV-2 (*p* ≤ 0.05) (Figures 11G,H). Also, percentages of memory CD44+ CD8+ T cells in co-cultures with DCs exposed to all NPs-HSV-2 were significantly increased in comparison DCs stimulated with inactivated HSV-2 (*p* ≤ 0.05) (Figure 11I). However, only treatment of DCs with all sizes of TA-AgNPs-HSV and L TA-AuNPs induced significantly higher percentages of CD44+ CD8+ T cells in comparison to HSV-2 infected control (*p* ≤ 0.05) (Figure 11I). Treatment with polymyxin B significantly blocked activation of T cells.

## Direct Effect of NPs and T Cells

**Figure 12:**
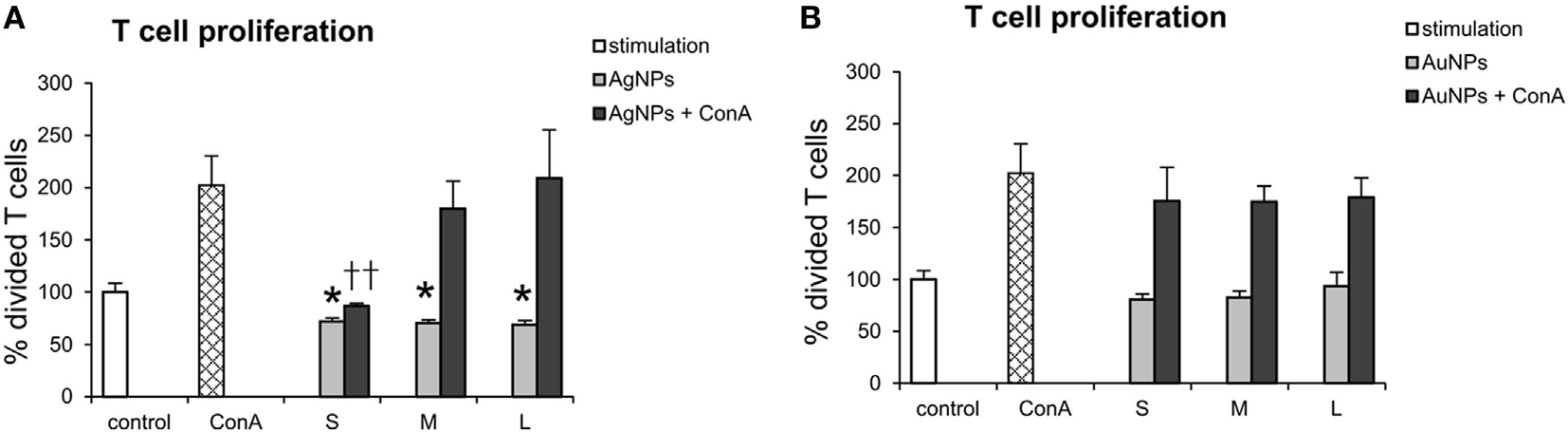
Direct effect of nanoparticles (NPs) on proliferation of T cells. T cell cultures with or without concanavalin A (ConA) were cultured with 2.5 µg/ml of **(A)** 10 nm (S), 37 nm (M), 59 nm (L) TA-AgNPs and **(B)** 10 nm (S), 34 nm (M), 62 nm (L) TA-AuNPs for 72 h. Each bar represents the mean from 3 experiments (*N* = 3) ± S.E.M. *Significant differences with *p* ≤ 0.01, ††*p* ≤ 0.01 calculated in relation to concanavalin A-treated control.

In our experiments with co-culture of DCs with T cells, the NPs were not present—they were washed out before addition of T cells. However, taking into account possibility of T cells coming into contact with NPs, we checked direct effects of TA-Ag/AuNPs on T cells. For T cells exposed to TA-modified 10 nm, 37 nm, and 59 nm AgNPs, the EC50 values were as follows: 11.22 ± 1.41 µg/ml, 17.85 ± 1.46 µg/ml, and 27.69 ± 4.77 µg/ml, respectively. For exposure of T cells to TA-modified 10 nm, 34 nm, and 62 nm AuNPs, EC50 values were 29.03 ± 2.95 µg/ml, 16.69 ± 0.15 µg/ml and 17.93 ± 0.55 µg/ml, respectively. To access how direct contact with NPs may influence T cell proliferation, we cultured T cells for 72 h with or without concanavalin A in the presence of 2.5 µg/ml TA-Ag/AuNPs and accessed T cell proliferation. Our data show that although all sizes of TA-AgNPs significantly decrease spontaneous proliferation of T cell cultures (*p* ≤ 0.01) (Figure 12A), only 10 nm TA-AgNPs can significantly decrease proliferation induced by concanavalin A (*p* ≤ 0.01) (Figure 12A). No significant influence of TA-AuNPs upon spontaneous or concanavalin-induced proliferation was observed (Figure 12B).

## Discussion

Taking into account an increasing interest in NPs as a new class of microbicides, the aim of this work was to check whether anti-viral activity of TA-AgNPs is followed by an adjuvant and immune-stimulatory effect. Various papers have demonstrated that metal NPs such as silver, gold, and iron oxide enhance the immunogenicity of antigens (9, 13, 20, 29). Therefore, apart from other applications of NPs, they can also be used as immune modulators. The material from which an NP is made as well as size, shape, and surface coating has a direct influence on the functions of APCs (9). Most studies use negatively charged citrate-coated NPs (9, 29), but the higher hydrophobicity of AuNP was shown to activate the innate immune system (TNF-α secretion) (30). Tannins are water-soluble phenol derivatives naturally synthesized and accumulated by higher plants as secondary metabolic products. Tannic acid (penta-m-digalloyl glucose) is the simplest and principal hydrolyzable tannin shown to exert anti-oxidative, anti-inflammatory, and antiviral properties (31, 32). It is known that the polyphenolic nature of tannic acid (hydrophobic core and hydrophilic shell) is the feature responsible for its interaction with cellular surface proteins (33). In our previous work, we showed that TA-AgNPs induced production of cytokines and chemokines by keratinocytes and macrophages (16), but also decreased inflammatory response induced by TNF-α and LPS (17). Additionally, TA-AgNPs directly interact with the surface of HSV-2 leading to a significant decrease in viral titers during both *in vitro* and *in vivo* infections (15). Treatment of the HSV-2 infected mucosal tissue with TA-AgNPs resulted not only in the decreased viral titers (15) but also in higher anti-HSV-2 antibody titers during recovery stage as shown by seroneutralisation tests (unpublished data). For HSV-2, the submucosal DCs present viral antigens to CD4+ T cells in the draining lymph nodes and help to generate the protective immune responses (28). HSV-2 treated in the mucosal tissue with TA-AgNPs may be effectively internalized by migratory DCs and activate them to present and prime CD4+ T cells.

Therefore, we used TA-AgNPs and -AuNPs of similar sizes to compare their immune-stimulatory and adjuvant effects in JAWS II mouse DCs exposed to TA-Ag/AuNPs, but also to NPs-treated HSV-2. This cell line has stable characteristics of DCs and is able to grow at high density without limitations of BMDCs cultures (34).

Gold nanoparticles are inert and non-toxic, while AgNPs are known to release silver ions, which can further influence cellular metabolism (35–39). Both types of NPs can be easily taken up by DCs and other phagocyting cells leading to their activation. As shown previously, toxicity of NPs modified with tannic acid can vary with the cell type, cellular uptake, and size (16, 17). Here, we show that TA-AgNPs and TA-AuNPs also demonstrate material- and size-dependent toxicity against JAWS II cells with AgNPs being more toxic than AuNPs. Similar tendency was observed in HECa10 cells, which showed relatively small toxicity starting from 5 µg/ml, proving TA-NPs > 30 nm safety for the local use such as injections. These results are consistent with previous reports (38, 39) and the data showed for RAW 264.7 mouse monocytic cell line exposed to TA-AuNPs (40). Toxicity of TA-AuNPs was previously observed for concentrations starting from 10 µg/ml (40). Postulated by Park et al. the Trojan-horse mechanism of AgNPs cytotoxicity involves release of silver ions from the surface of NPs in acidified compartments. The released ions induce ROS production triggering activation of immune cells through stress signals and secreted cytokines (i.e., TNF-α) (39). In our study, all TA-AgNPs showed reduced sizes in TEM images in comparison to their initial dimensions, indicating partial decomposition of TA-AgNPs by JAWS II cells and release of silver ions. Interestingly, when compared with the toxicity observed in RAW 264.7 mouse monocyte line exposed to TA-AgNPs, the same NPs demonstrate lower toxicity against DC line and no production of TNF-α (16). This may be due to a lower acidification of compartments occupied by NPs leading to a lower release of silver ions in DCs.

Nanoparticles have been shown to enter the cell via four types of pathways: clathrin/caveolar-mediated endocytosis, phagocytosis, macropinocytosis, and pinocytosis. JAWS II DCs treated with both AgNPs and AuNPs demonstrated activated morphology with numerous microvilli and funnel configured lamellipodia decorated with NPs, characteristic for macropinocytosis and endocytosis (41, 42). Since JAWS II express receptors for negatively charged molecules (i.e., DEC-205) (42) and all tested NPs have negative charge, JAWS II cells utilize receptor-mediated endocytosis for NP internalization, a pathway important for DC activation (41, 42). Furthermore, we found that inhibitor of clathrin-mediated endocytosis blocked uptake of TA-Ag/AuNPs. Clathrin- and caveolin-mediated endocytosis and phagocytosis are believed to be typical pathways for uptake of NPs by actively phagocyting cells such as DCs (43). After 24 h exposure, NPs were localized mainly within electron-dense vacuoles attached to the inner vacuole membrane. Such pattern was also observed by Yen et al., but only for AuNPs (44). Internalization of TA-Ag/AuNPs was also influenced by CChD, an inhibitor of phagocytosis and macropinocytosis. The macropinocytosis pathway is a non-specific process to internalize fluids and particles together into the cell. The phagocytosis pathway is actin-dependent and restricted to professional phagocytes, such as macrophages, DCs, and neutrophils. Phagocytosis is used to internalize particles bigger than 0.5 µm. Most NPs tend to aggregate in biological solutions, increasing their overall size. Thus, we cannot exclude that tannic acid-modified NPs used in this study form aggregates in biological fluids. However, by forming aggregates with viral antigens, tannic acid-modified NPs may help to internalize viral antigens and help to present them to immune competent cells.

Efficient antigen presentation requires presence of the MHC class I or II, but also a second signal from the co-stimulatory molecules (45). Here, JAWS II exposed to non-toxic doses of all tested NPs decreased MHC class I expression and up-regulated MHC class II. The MHC II molecules are essential for the antigen presentation by APC to the naive T cell, which is followed by the activation of the adaptive immune response. Xu et al. (13) showed that non-toxic doses of AgNPs up-regulated MHC II expression on murine macrophages, while in the study by Tomić et al. (46) unmodified AuNPs of 10 and 50 nm had no effect upon CD83, CD86, and MHC II activation and suppressed LPS-induced up-regulation of MHC II, CD83, and CD86. When an NP enters a biological environment, it comes into contact with a biofluid that contains a diverse mixture of proteins. A subset of these proteins will adsorb to its surface, forming a protein “corona” (47). It is believed that composition of this corona largely defines the biological identity and activity of the particle (47). In this study, we used tannic acid-modified NPs, which can result in different composition of the protein corona and thus different reaction of immune competent cells, in comparison to unmodified AgNPs or AuNPs. Interestingly, stimulation of JAWS II cells with all tested TA-AgNPs increased expression of CD40 and CD86, while only 10 nm AuNPs increased CD86 expression. In contrast, all tested NPs down-regulated expression of CD80. The experiments with endocytosis and phagocytosis inhibitors showed that contact with the surface of tannic acid-modified NPs is important for early down-regulation of MHC I expression but does not lead to early activation of DCs. Internalization of TA-Au/AgNPs is clearly important for further activation of DCs. Therefore, we can hypothesize that later changes in CD40, CD80, CD86, and MHC II expression and activation status is related with the metal type and size and requires NPs internalization. As mentioned above, AgNPs can undergo decomposition in acid cellular compartments and thus influence DCs activation.

The follow-up experiments with BMDCs cultures obtained from C57BL/6 mice generally followed the pattern observed for JAWS II cells (Figure S1 in Supplementary Material). However, we did not detect any differences for CD86 expression and a high distribution of individual results for CD80. This confirms utility of JAWS II cell line for studies of DCs biology.

Previously, we have shown that TA-AgNPs exert a size-dependent inhibition of infection by blockage of HSV-2 attachment and penetration, with small NPs being the most effective (15). JAWS II cells exposed to HSV-2 treated with 10 and 37 nm TA-AgNPs internalized HSV-2 antigens more efficiently than live or inactivated HSV-2. Here, the NPs are modified with tannic acid, which further increases their interaction with proteins and apparently helps to form aggregates with viral proteins, which are further internalized. Furthermore, silver NPs better exposed viral antigens to host receptors than AuNPs of the same size, which suggest the effect of the silver upon antigens internalization. This is in contrast to the paper by Xu et al. reporting that AgNPs did not act as a cargo for the cellular delivery of the antigens to macrophages, and the adjuvant effect of AgNPs was mainly ascribed to the recruitment and activation of local leukocytes (13). Again, AgNPs used by Xu et al. are unmodified NPs (13).

Toll-like receptors belong to the pattern-recognition receptors (PRRs) and are crucial during recognition of pathogens by DCs to trigger adaptive immune response (27, 48). HSV-2 is detected by PRRs through glycoproteins, RNA, and genomic DNA (49). TLR9 is expressed mainly within endosomal compartment and recognizes CpG motifs in viral DNA and it has been demonstrated that it is involved in HSV recognition (49). Response to CpG-DNA recognized by TLR9 leads to up-regulation of MHC class II and CD40, CD80, and CD86 co-stimulatory molecules (27, 48). In this study, elevated levels of TLR9 mRNA were found during stimulation with all TA-AgNPs and 34 nm TA-AuNPs alone. Only TA-AgNPs-treated HSV-2 was able to activate TLR9 expression indicating that AgNPs rather than AuNPs allow for better recognition of viral DNA.

While HSV-2 infection of DCs inhibits their maturation and thus results in a poor development of the specific immune response, inactivated HSV-2 has also been shown to be a poor antigen for immature DCs (19). In our studies, HSV-2 infection of JAWS II mouse DC line resulted in down-regulation of CD40 and MHC class II but not CD86 or CD80, while inactivated HSV-2 had little influence on activation of DCs. As shown in Figure 9, both AgNPs and AuNPs can help to present viral antigens through MHC class II, while AgNPs can help to cross-present viral antigens to CD8+ T cells though stimulation of MHC I expression. HSV-2 treated with TA-AgNPs of all sizes and small TA-AuNPs were better stimulants of MHC I expression than bigger TA-AuNPs. MHC class I is present on all nuclear cells presenting self-antigens. However, DCs are able to cross-present captured antigens in the context of MHC class I to CD8+ T-cells, which is of importance for anti-HSV-2 response (49–51). Furthermore, all preparations of HSV-2 treated with NPs up-regulated expression of MHC class II in comparison to cells infected with live or inactivated virus.

Furthermore, NPs can also help to overcome inhibition of DCs maturation by live or inactivated virus due to up-regulation of co-stimulatory molecules, necessary for effective antigen presentation. All NPs-treated HSV-2 retained CD86 expression and down-regulated CD80 expression in comparison to infection with live HSV-2. Down-regulation of CD40 expression in HSV-2 infection of DCs was overcome when HSV-2 was treated with NP sized >30 nm. Ligation of PD-L1 to its receptor PD-1 inhibits activation and expansion of CD8+ T cells, rendering them dysfunctional. Infection of DCs with live HSV-2 leads to an up-regulation of PD-L1 expression, as shown by Krzyzowska et al. (52). Here, PD-L1 expression was conversely related with TA-AgNPs size, while an opposite effect was observed for TA-AuNPs.

Interestingly, TA-AgNPs-treated HSV-2 elicited significantly lower TNF-α inflammatory response as well as decreased IL-6 production in comparison to live HSV-2 (Figure 10). Reduced secretion of IL-6 during antigen presentation may lead to development of CD4+ Th2-dependent response which is important during infection of mucous membranes (53). It has been shown that high titers of specific anti-HSV antibodies decrease the risk of recurrent intravaginal infection (54).

Co-culture of HSV-2 infected JAWS II cells with T cells obtained from UV-HSV-2 challenged mice confirmed inhibitory effects of HSV-2 infection on JAWS II maturation. HSV-2 infected DCs showed the lowest ability to stimulate proliferation of antigen-specific T cells in comparison to heat-inactivated HSV-2. In contrast, treatment of HSV-2 with all tested NPs stimulated JAWS II DCs to induction of T cell proliferation. We can therefore conclude that tannic acid NPs-treated HSV-2 help to present viral antigens to antigen-specific memory T cells and effectively activate both CD4+ and CD8+ T cells. Upon T cells activation, CD69 is usually expressed only up-to 48 h, then rapidly down-regulated. However, we still observed up-regulation of CD69 on T cells after 72 h of co-culture with NPs-HSV stimulated JAWS II. These data are in agreement with those obtained by Fazekas De St Groth showing that T cells destined to divide will express CD69 (55). Furthermore, co-cultures of antigen-specific T cells with DCs stimulated with NPs-treated HSV-2 showed that TA-AgNPs of all sizes and only large TA-AuNPs lead to significant increase in CD4+ IFN-γ+ cells, indicating that TA-NPs can help to stimulate Th1 response. This was followed in this study by a significant increase in CD8+ IFN-γ+ T cells, important for cytotoxic response to virus-infected cells. Additionally, we found that all TA-NPs were better stimulants of memory CD8+ T cells in comparison to inactivated HSV-2, while all AgNPs and only large AuNPs were better stimulants of memoryCD8+ T cell proliferation than live HSV-2. Recent study by Srivastava et al. showed that mobilization of functional protective memory CD8+ T cells within the site of infection protects from acute and recurrent genital herpes infection (56). CD8+ T cell response to HSV infection requires CD4+ T cells and CD40L-CD40 interactions with DCs cells (57). The presence of polymyxin B sulphate influenced induction of CD40 expression on DCs upon TA-NPs-HSV-2 treatment. Since this antibiotic consists of a cyclic heptapeptide tripeptide side chain and a fatty acid tail, it can interact with hydrophobic moieties of tannic acid. However, the exact character of polymyxin B–TA-NPs-HSV-2 interaction is unknown and probably complex.

Interestingly, TA-Ag/AuNPs added directly to T cell cultures, had no effect upon proliferation of activated T cells, except for 10 nm AgNPs. This is in contrast to the results of work by Devanabanda et al., where gold and silver sized 30 and 60 nm significantly affected concanavalin A stimulated responses of murine splenic lymphocytes (58). However, concentrations used in this study were approximately 10 times lower. This indicates that TA-AgNPs or TA-AuNPs do not inhibit activated T cells.

For HSV-2, the pathway to antigen presentation is complex involving multiple types of DCs. The virus first infects LCs, which undergo apoptosis and are taken up by bystander DCs. The migratory DCs carry HSV antigen out of the tissue and are essential for T-cell priming in the lymph node (28). Taking into account the use of TA-AgNPs as microbicides, we can therefore conclude that upon treatment of primary infection, AgNPs-treated HSV-2 may be effectively internalized by migratory DCs, activate them to present and prime CD4+ and CD8+ T cells. During secondary infection, NPs-treated HSV-2 antigens can effectively activate antigen-specific memory T cells.

We found TA-AgNPs as better stimulators of DCs maturation than TA-AuNPs which is probably due to their higher bioactivity. There are also many studies showing that AgNPs directly stimulate the innate immune system (30, 47). Another possibility is that both metals activate different cellular pathways related with antigen presentation and DC activation as shown here for expression of cytokines and TLR9. Furthermore, we also showed for the first time that tannic acid modification of AgNPs indeed helped to internalize antigens.

In summary, increased immunogenicity of NPs-treated HSV-2 may contribute not only to a faster healing of mucous membrane infection after treatment with TA-AgNPs but also for better development of specific immune response which may cause higher resistance to recurrent infection. Since mucous membranes are attractive targets for vaccines and AgNPs or AuNPs are relatively biocompatible, we can speculate that our results may provide data for preliminary studies on nano-metal-based class of nano-adjuvants.

## Ethics Statement

This study was performed in strict accordance with the recommendations of the Polish Act of 21 January 2005 on animal experiments (OJ no. 33, item 289) and Directive 2010/63/EU of the European Parliament and the Council of 22 September 2010 on the protection of animals used for scientific purposes. The protocol was approved by the 4th Local Committee on the Ethics of Animal Experiments in Warsaw, Poland (Permit Number: 51/2013).

## Author Contributions

PO, MK, GC, and JG contributed conception and design of the study; PO, ET, KR-S, TM, and GC performed lab experiments; MG, OL, and JN performed TEM analysis; GC performed confocal imaging; PO wrote the first draft of the manuscript; ET, KR-S, GC, JG, MK, TM, MG, OL, and GC wrote sections of the manuscript. All the authors have read and approved of the final manuscript.

## Conflict of Interest Statement

The authors declare that the research was conducted in the absence of any commercial or financial relationships that could be construed as a potential conflict of interest.
