# Supplementary material for: Tannic Acid-Modified Silver and Gold Nanoparticles as Novel Stimulators of Dendritic Cells Activation
Source: Front Immunol. 2018 May 22;9:1115. doi: 10.3389/fimmu.2018.01115 (PMC5972285; doi:10.3389/fimmu.2018.01115)
Supplement: Supplementary file 1 [file image_1.PDF]

Supplementary Material

Tannic acid-modified silver and gold nanoparticles as novel stimulators of dendritic cells activation

Piotr Orlowski, Emilia Tomaszewska, Katarzyna Ranoszek-Soliwoda, Marianna Gniadek, Olga Labedz, Tadeusz Malewski, Julita Nowakowska, Grzegorz Chodaczek, Grzegorz Celichowski, Jaroslaw Grobelny, Malgorzata Krzyzowska

*** Correspondence:** Corresponding Author: krzyzowskam@yahoo.com

Supplementary Figure 1. Effect of NPs on expression of surface activation markers in bone-marrow derived dendritic cells (BMDCs). MHC class I (A), MHC class II (B), CD40 (C), CD86 (D) and CD80 (E) expression on the BMDCs after 24 h exposure to 2.5 μg/ml 10 nm (S), 37 nm (M), 59 nm (L) TA-AgNPs and 10 nm (S), 34 nm (M), 62 nm (L) TA-AuNPs. Representative histogram plots for MHC class I (F) and MHC class II (G), CD40 (H), CD86 (I) and CD80 (J). Each bar represents the mean from 3 experiments (N = 3) ± S.E.M., * represents significant differences with $p \leq 0.05$, while ** means $p \leq 0.01$

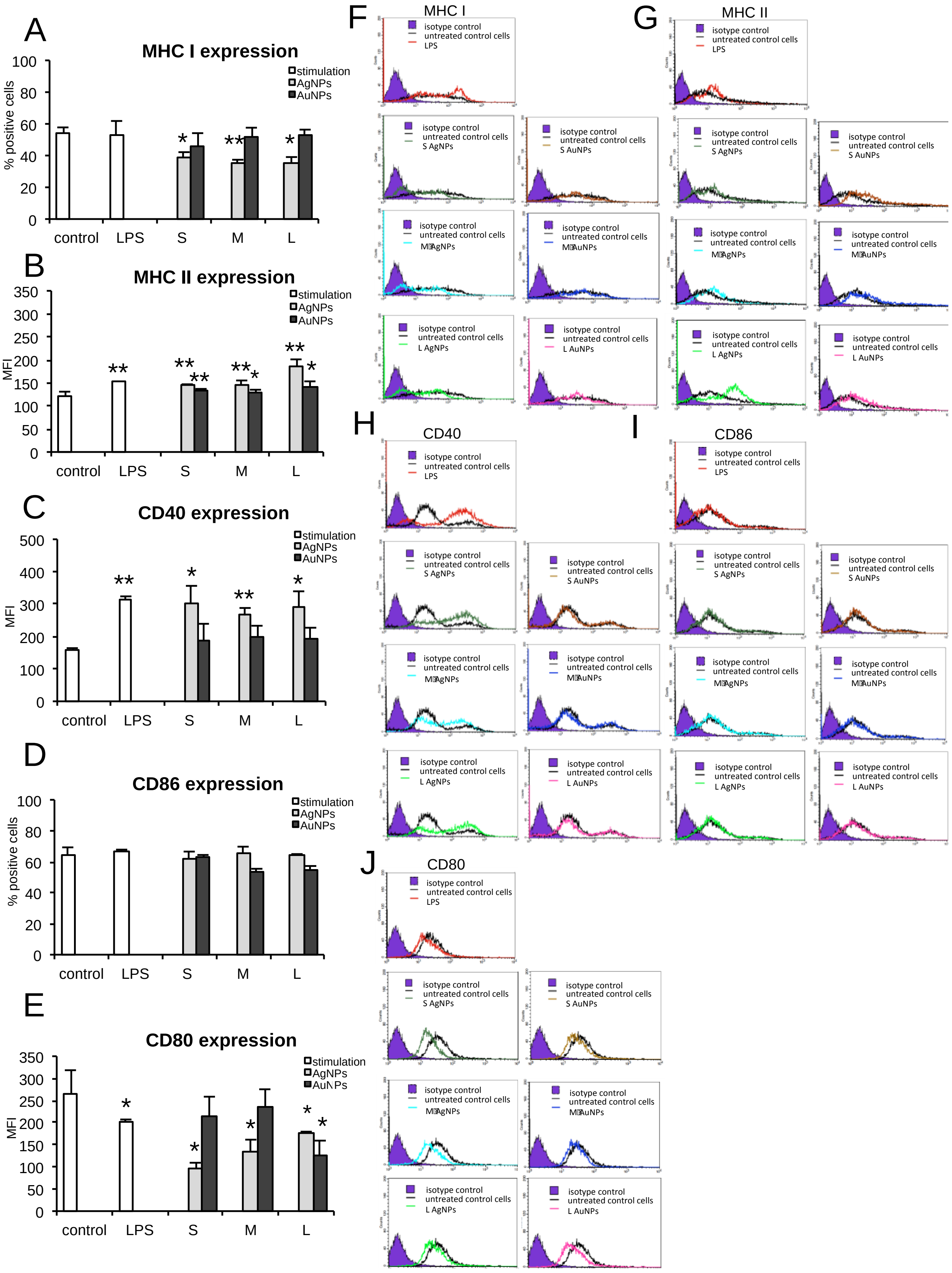
A
MHC I expression
stimulation
AgNPs
AuNPs
% positive cells
control
LPS
S
M
L
B
MHC II expression
MFI
C
CD40 expression
D
CD86 expression
E
CD80 expression
F
MHC I
G
MHC II
H
CD40
I
CD86
J
CD80
isotype control
untreated control cells
LPS
S AgNPs
S AuNPs
L AgNPs
L AuNPs
