# Supplementary material for: Tannic Acid-Modified Silver and Gold Nanoparticles as Novel Stimulators of Dendritic Cells Activation
Source: Front Immunol. 2018 May 22;9:1115. doi: 10.3389/fimmu.2018.01115 (PMC5972285; doi:10.3389/fimmu.2018.01115)
Supplement: Supplementary file 2 [file table_1.PDF]

Supplementary Material

Tannic acid-modified silver and gold nanoparticles as novel stimulators of dendritic cells activation

Piotr Orlowski, Emilia Tomaszewska, Katarzyna Ranoszek-Soliwoda, Marianna Gniadek, Olga Labedz, Tadeusz Malewski, Julita Nowakowska, Grzegorz Chodaczek, Grzegorz Celichowski, Jaroslaw Grobelny, Malgorzata Krzyzowska

*** Correspondence:** Corresponding Author: krzyzowskam@yahoo.com

Supplementary Table 1. Cytotoxicity assays in endothelial cells from mouse peripheral lymph nodes (HECa10) at 24 h of exposure to AgNPs sized 10 nm, 37 nm, 59 nm and AuNPs sized 10 nm, 34 nm, 62 nm at 0.5-10 µg/ml. HECa10 mouse endothelial cell line was kindly provided by Professor Claudine Kieda from the Centre of Molecular Biophysics, Orléans, France and grown in DMEM supplemented with 10% HI-FBS, 100 U/ml penicillin and 100 µg/ml streptomycin (Gibco) in standard conditions. Prior tests cells were seeded into 96-well plates at the density of 10^4 per well for 24 h. Next, medium was discarded and replaced with fresh medium containing different concentration of nanoparticles. The Neutral red test was performed as described previously (16). The viability of cells was expressed as a percentage of the control, untreated cells (100%). Table presents means from 3 experiments (N = 3) ± S.E.M., * represents significant differences with p ≤ 0.05, while ** means p ≤ 0.01.

	10 TA-AgNPs	37 nm TA-AgNPs	59 nm TA-AgNPs	10 nm TA-AuNPs	34 nm TA-AuNPs	62 nm TA-AuNPs
1 µg/ml	89.04 ± 7.15%	92.97 ± 2.22%	115.67 ± 4.44%	108.67 ± 16.06%	80.66 ± 0.66 %	91.02 ± 1.68 %
2.5 µg/ml	79.26 ± 4.32%*	89.88 ± 4.03%	98.57 ± 10.89%	95.02 ± 2.94%	81.47 ± 4.93 %	96.55 ± 1.57 %
5 µg/ml	73.81 ± 1.04%**	78.71 ± 4.87%	92.71 ± 1.69%	80.81 ± 0.94%*	87.04 ± 4.69 %	85.75 ± 2.29 %
10 µg/ml	58.22 ± 2.23%**	45.28 ± 4.29%**	76.94 ± 3.48%**	73.33 ± 6.24%**	76.24 ± 2.29 %**	66.81 ± 5.56 %**
