# Supplementary material for: Tannic Acid-Modified Silver and Gold Nanoparticles as Novel Stimulators of Dendritic Cells Activation
Source: Front Immunol. 2018 May 22;9:1115. doi: 10.3389/fimmu.2018.01115 (PMC5972285; doi:10.3389/fimmu.2018.01115)
Supplement: Supplementary file 3 [file table_2.PDF]

Supplementary Material

Tannic acid-modified silver and gold nanoparticles as novel stimulators of dendritic cells activation

Piotr Orlowski, Emilia Tomaszewska, Katarzyna Ranoszek-Soliwoda, Marianna Gniadek, Olga Labedz, Tadeusz Malewski, Julita Nowakowska, Grzegorz Chodaczek, Grzegorz Celichowski, Jaroslaw Grobelny, Malgorzata Krzyzowska

*** Correspondence:** Corresponding Author: krzyzowskam@yahoo.com

Supplementary Table 2. CD40 expression on the JAWS II cells after 24 h exposure to HSV-2 treated with 2.5 µg/ml of 10 nm (S), 37 nm (M), 59 nm (L) TA-AgNPs and 10 nm (S), 34 nm (M), 62 nm (L) TA-AuNPs in the presence of polymyxin B (PmB). Table presents means from 3 experiments (N = 3) ± S.E.M., * represents significant differences with $p \leq 0.05$ in comparison to cells non-treated with PmB.

Stimulation	(-)	polymyxin B
control	209.36 ± 20.17	201.01 ± 15.2
HSV-2	122.26 ± 5.41	119.8 ± 5.45
inHSV-2	183.79 ± 15.95	189.2 ± 13.44
10 nm AgNPs + HSV-2	217.32 ± 20.52	187.45 ± 7.66*
37 nm AgNPs + HSV-2	209.07 ± 24.74	186.33 ± 9.34*
59 nm AgNPs + HSV-2	232.59 ± 23.44	193.5 ± 8.56*
10 nm AuNPs + HSV-2	162.73 ± 22.84	151.26 ± 11.9
34 AuNPs + HSV-2	167.42 ± 4.17	157. ± 14.21
62 AuNPs + HSV-2	172.01 ± 12.69	143.12 ± 13.45*
